# *Razanandrongobe sakalavae,* a gigantic mesoeucrocodylian from the Middle Jurassic of Madagascar, is the oldest known notosuchian

**DOI:** 10.7717/peerj.3481

**Published:** 2017-07-04

**Authors:** Cristiano Dal Sasso, Giovanni Pasini, Guillaume Fleury, Simone Maganuco

**Affiliations:** 1 Museo di Storia Naturale di Milano, Milano, Italy; 2 Appiano Gentile, Como, Italy; 3 Muséum d’Histoire Naturelle de Toulouse, Toulouse, France

**Keywords:** Cranial anatomy, Pachydont dentition, Mesoeucrocodylia, Notosuchia, Middle Jurassic, Madagascar

## Abstract

*Razanandrongobe sakalavae* Maganuco, Dal Sasso & Pasini, 2006 is a large predatory archosaur from the Middle Jurassic (Bathonian) of the Mahajanga Basin, NW Madagascar. It was diagnosed on the basis of teeth and a fragmentary maxilla, but its affinities were uncertain. Here we describe new cranial remains (above all, an almost complete right premaxilla and a caudally incomplete left dentary) that greatly improve our knowledge on this enigmatic species and reveal its anatomy to be crocodylomorph. The right premaxilla indicates that the rostrum was deep, wide, and not pointed; it bears five teeth that are sub-vertical and just slightly curved lingually; the mesial teeth are U-shaped in cross-section and have serrated carinae on the lingual side; the *aperturae nasi osseae* (external bony nares) are confluent and face rostrally; and there is no lateral groove at the premaxillomaxillary suture for reception of a hypertrophied lower caniniform tooth. The preserved portion of the left dentary has an edentulous tip and bears eight large mandibular teeth of which the mesial (1–3) are the largest, but none is a hypertrophied caniniform tooth; the mandibular (dentary) symphysis extends caudally to the level of the third tooth; the splenial is not preserved, but its sutural marks on the dentary indicate that it contributed to the mandibular symphysis for at least 20% of the symphyseal length in dorsal aspect. On the basis of this new data, some previously uncertain features of the holotype maxilla—such as the margin of the suborbital fenestra, the contact surfaces for the palatine, the ectopterygoid, and the jugal—are now apparent. Testing of the phylogenetic position of the species within Crocodylomorpha indicates that *R. sakalavae* is a mesoeucrocodylian. It also represents one of the earliest events of exacerbated increase in body size along the evolutionary history of the group. In addition, it is by far the oldest notosuchian. A cranial reconstruction of this gigantic predator is also attempted here. The very robust jaw bones of *R. sakalavae*, coupled with its peculiar dentition, strongly suggest a diet that included hard tissue such as bone and tendon.

## Introduction

A decade ago, Maganuco, Dal Sasso & Pasini (2006) described the fragmentary remains of a very large predatory archosaur from the Middle Jurassic (Bathonian) of the Mahajanga Basin, Madagascar. The material included a fragmentary right maxilla bearing three teeth, and seven peculiar isolated teeth clearly belonging to the same taxon. In spite of the scanty remains, the presence of a unique combination of features, which included a well-developed bony palate on the maxilla, mesial and lateral teeth respectively U-shaped and sub-oval in cross-section, and very large tooth denticles (1 per mm) on the carinae, allowed the authors to erect the new taxon *Razanandrongobe sakalavae* Maganuco, Dal Sasso & Pasini, 2006. However, the systematic position of the new species remained uncertain: indeed, besides the autapomorphic denticle size, *R. sakalavae* shared a mix of potential autapomorphic, synapomorphic, and homoplasic features with crocodylomorphs and theropods. Therefore, the species was referred to Archosauria *incertae sedis*.

Here we describe new cranial material referable to *R. sakalavae* and consisting of an almost complete right premaxilla, the rostral half of a left dentary, a maxillary fragment with diagnostic teeth, and a very large isolated tooth crown. In addition, we tentatively refer to the same taxon five cranial fragments that were likely collected at the same locality. This new material greatly improves our knowledge on the cranial anatomy of this species, permitting us to: (1) clarify some previously uncertain features of the holotype due to its fragmentary nature; (2) make more in-depth anatomical comparisons with members of Crocodylomorpha and Theropoda, definitely ruling out it pertaining to the latter group; (3) test the phylogenetic relationships of the species and shed light on the evolutionary history and paleobiogeography of Notosuchia; (4) attempt a cranial reconstruction; and (5) confirm previous remarks on its paleobiology.

## Material and Methods

**Figure 1:**
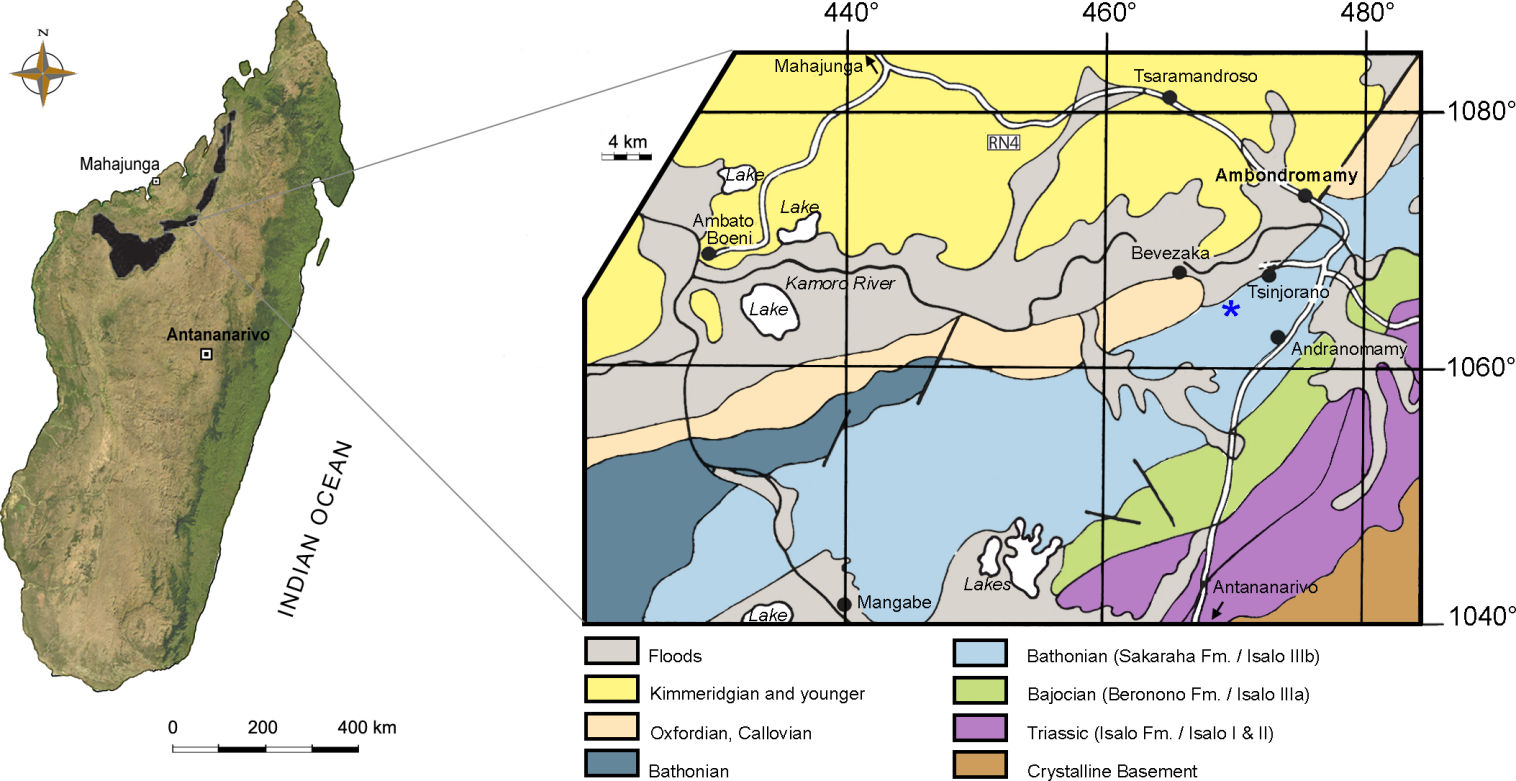
Map of Madagascar. Map depicting the Triassic and Jurassic outcrops of the Mahajanga Basin (black areas on the left), and geological map of the surroundings of Ambondromamy, highlighting the Sakaraha Fm. (light blue). Part of the material described herein (the premaxilla and the dentary) comes from the area marked by the blue asterisk. Based on Besairie (1968–1969).

The most relevant material described here consists of an almost complete right premaxilla, the rostral half of a left dentary, and a very large isolated tooth crown. The first two specimens belong to the same individual (see below) and are deposited at the Muséum d’Histoire Naturelle de Toulouse under catalogue numbers MHNT.PAL.2012.6.1 (dentary) and MHNT.PAL.2012.6.2 (premaxilla). They were collected by the assistant director of technical services of Société Sucrière de la Mahavavy (D Descouens, pers. comm., 2012) between 1972 and 1974 in the surroundings of Ambondromamy (Fig. 1), the same locality of the Mahajanga Basin that yielded the holotype of *R. sakalavae* (Maganuco, Dal Sasso & Pasini, 2006) and the sauropod *Archaeodontosaurus* (Buffetaut, 2005). The specimens were exported from Madagascar under authorization No. 1702 and 2547 of the Mines and Energy Directorate, Ministry of Economy and Finance. Recent careful preparation allowed the specimens to be recognized as belonging to the enigmatic species *Razanandrongobe sakalavae*. In April 2012, they were acquired by the Muséum d’Histoire Naturelle de Toulouse from the private collection of D Descouens, together with six other cranial fragments anatomically reminiscent of *R. sakalavae* (catalogued as MHNT.PAL.2012.6.3 to 8) that were collected from an indeterminate Malagasy locality. Of the latter, the largest bone pieces are spongier and more friable, with residual patches of smoothed matrix (possibly due to recent chemical preparation); the smallest pieces are proportionally heavier, denser, whitish, and polished (which suggests prolonged exposure to air and sunlight).

The very large isolated tooth crown (catalogue n. MSNM V7144) is part of the fossil vertebrate collection at the Museo di Storia Naturale di Milano. This specimen was collected several years ago in the Mahajanga Basin by G Cortenova, an Italian agronomist living in Madagascar. Before his death, Cortenova gave it to G Colombo, an amateur entomologist, who eventually donated the specimen to the museum.

The matrix encrusting the nasal attachment of the premaxilla was removed, analyzed, and compared to the matrix of the holotype maxilla. They were found to be similar in aspect and mineralogical composition, supporting the hypothesis that the three specimens came from the same geological age [i.e., Middle Jurassic, Bathonian,167.7–164.7 MA (Cohen et al., 2013)] and stratigraphic horizon [Sakaraha Formation *sensu* Geiger, Clark & Mette (2004), formerly mentioned in the literature as the Isalo IIIb subunit,‘Faciès Mixte Dinosauriens’ (Besairie, 1972)].

Measurements were taken with digital calipers. For the teeth, we used the following parameters: TCH, tooth crown height; FABL, fore-aft basal length; BW, basal width; BCR (FABL/BW), basal compression ratio; ER (FABL/TCH), elongation ratio [all these parameters are from Currie, Rigby Jr & Sloan (1990) and Farlow et al. (1991)]; and DSDI, denticle size difference index (Rauhut & Werner, 1995). Where possible, denticle count was taken at mid-crown height because the size of the denticles decreases at the apical and basal ends of both carinae. The systematic terms are taken mainly from Brochu (2001) and Pol et al. (2014). Additional systematic terms concerning the relationships among theropods, basal Crurotarsi, and mesoeucrocodylians are taken respectively from Weishampel, Dodson & Osmólska (2004), Parrish (1993) and Sereno et al. (2001).

**Figure 2:**
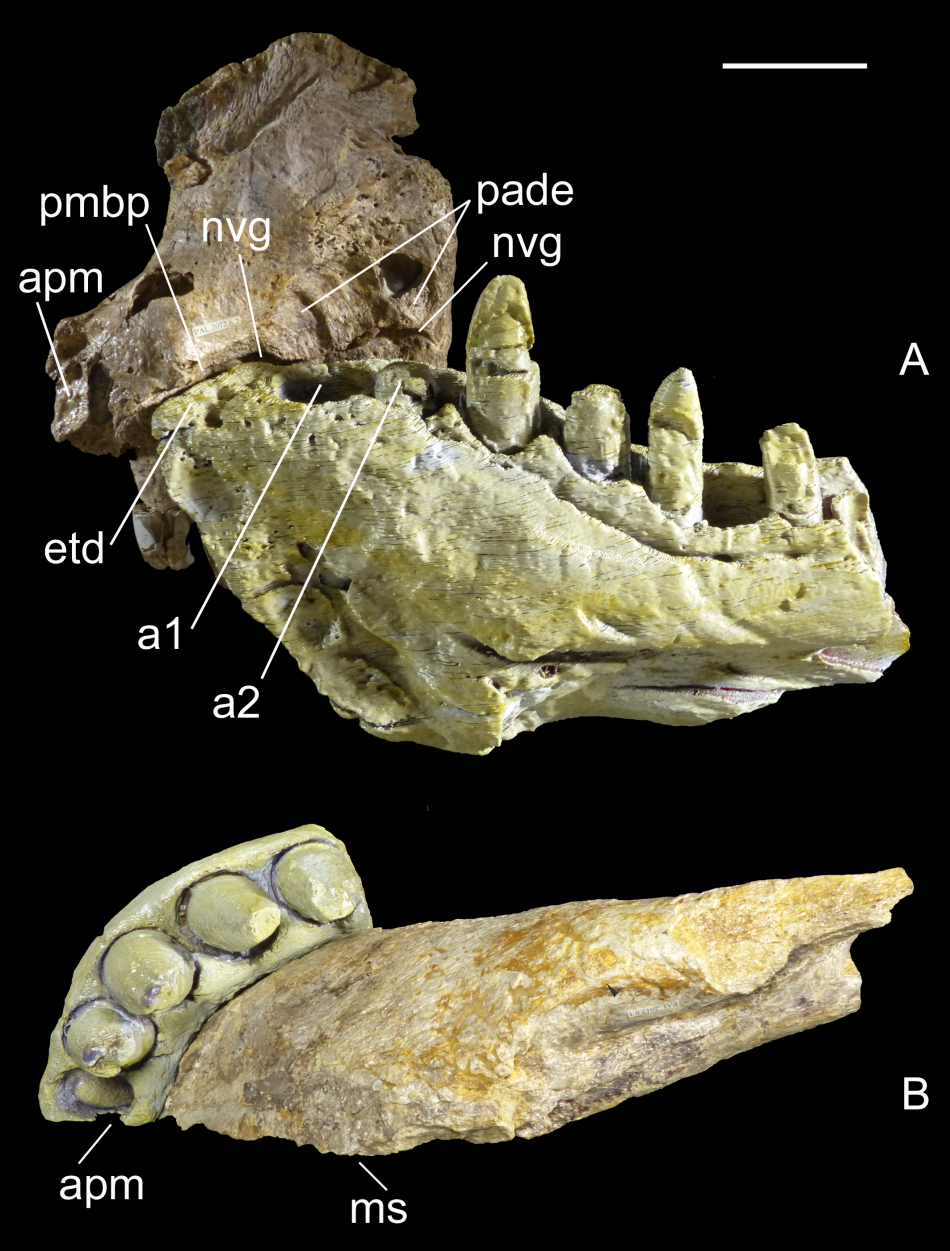
Two bones, one individual. Identical counterlateral copies, 3-D printed from CT data, of the left dentary (MHNT.PAL.2012.6.1) and right premaxilla (MHNT.PAL.2012.6.2) herein described, rearticulated with the original specimens. The perfect occlusion of the two bones, in medial (A) as well as in ventral (B) views, unequivocally demonstrates that MHNT.PAL.2012.6.1 and MHNT.PAL.2012.6.2 pertain to the same individual. Scale bar = 5 cm. Abbreviations: see text.

Computed tomography (CT) of the two most important referred specimens was performed at the Radiology Department, Spedali Civili di Brescia, Italy, with a Siemens Dual Source Scanner SOMATOM Definition Flash CT. Analysis and post-processing were performed at Siemens Milano, Italy, with a SyngoVia post-processing system using the Region Growing Algorithm to segment volumes and visualize internal anatomical structures. The CT data of the right premaxilla and left dentary were also used to print life-size identical counterlateral bones in 3-D, permitting us to rearticulate them and verify their juxtaposition at jaws closed (Fig. 2). This revealed that the labial margin of the dentary perfectly fits the rim of the medial neurovascular groove of the premaxilla, and that the rostral tip of the dentary symphysis fits the descending margin of the interpremaxillary suture. Moreover, the premaxilla has a bony palate overhanging the edentulous tip of the dentary, leaving no space for any teeth, and two large notches just in front of the first and second dentary alveolus, which accommodated enormous teeth erupting from the dentary. In ventral view, the curvature of the labial sides of the two bones are identical. Moreover, the sutures of the counterlateral bones are correctly aligned along the medial sagittal plane. The perfect occlusion of the two bones demonstrates unequivocally that MHNT.PAL.2012.6.1 and MHNT.PAL.2012.6.2 pertain to the same individual.

### Systematic paleontology

**Table:** 

CROCODYLOMORPHA Walker, 1970 (*sensu* Clark, 1986)
CROCODYLIFORMES Hay, 1930 (*sensu* Clark, 1986)
MESOEUCROCODYLIA Whetstone & Whybrow, 1983 *sensu* Benton & Clark, 1988
NOTOSUCHIA Gasparini, 1971
*RAZANANDRONGOBE SAKALAVAE* Maganuco, Dal Sasso & Pasini, 2006

**Holotype**—MSNM V5770, a fragmentary right maxilla bearing three unerupted teeth (Maganuco, Dal Sasso & Pasini, 2006: figs. 4–6).

**Figure 3:**
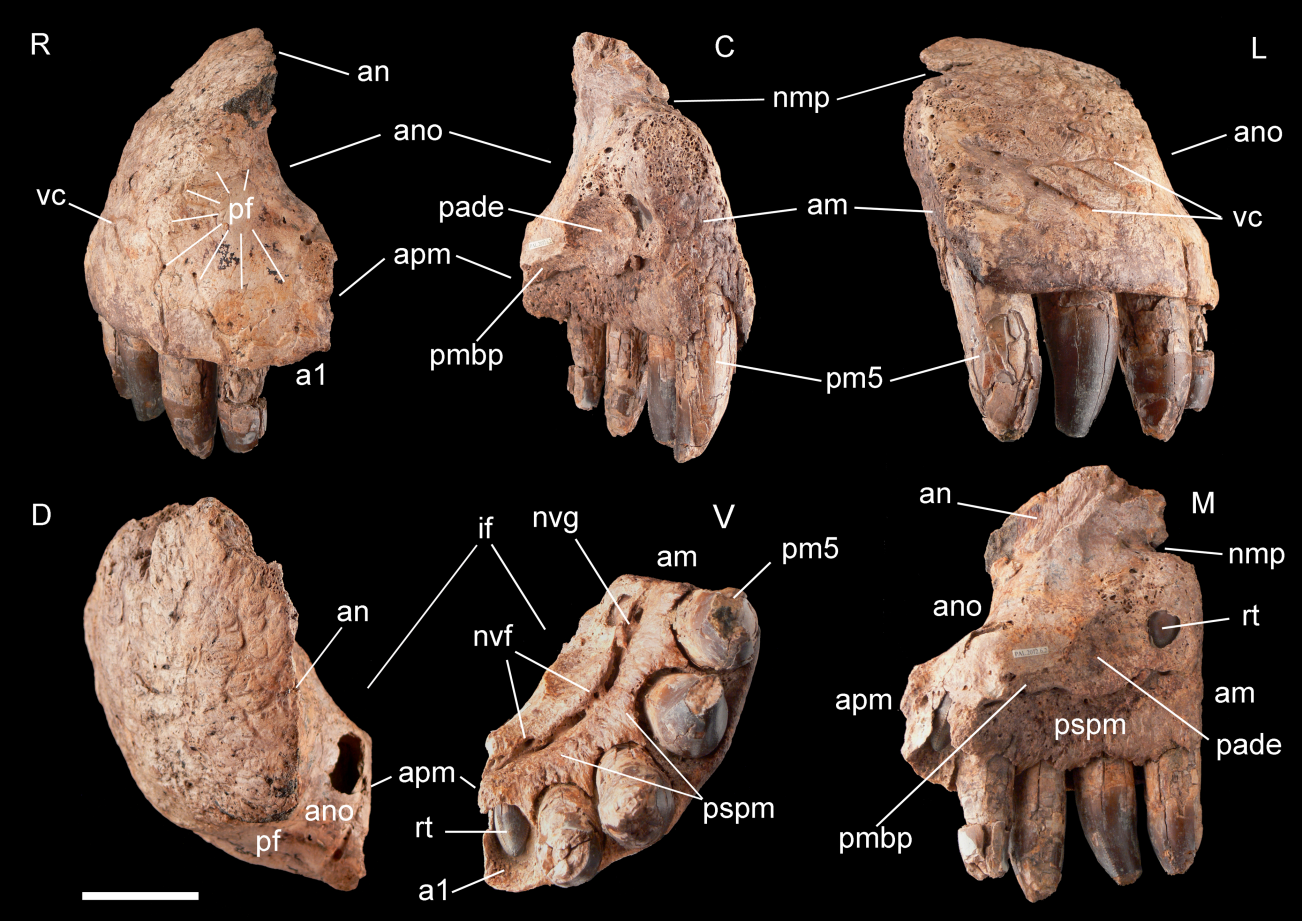
Right premaxilla of *Razanandrongobe sakalavae*. Specimen MHNT.PAL.2012.6.2 in rostral (R), caudal (C), lateral (L), dorsal (D), ventral (V), and medial (M) views. Scale bar = 5 cm. Abbreviations: see text.

**Figure 4:**
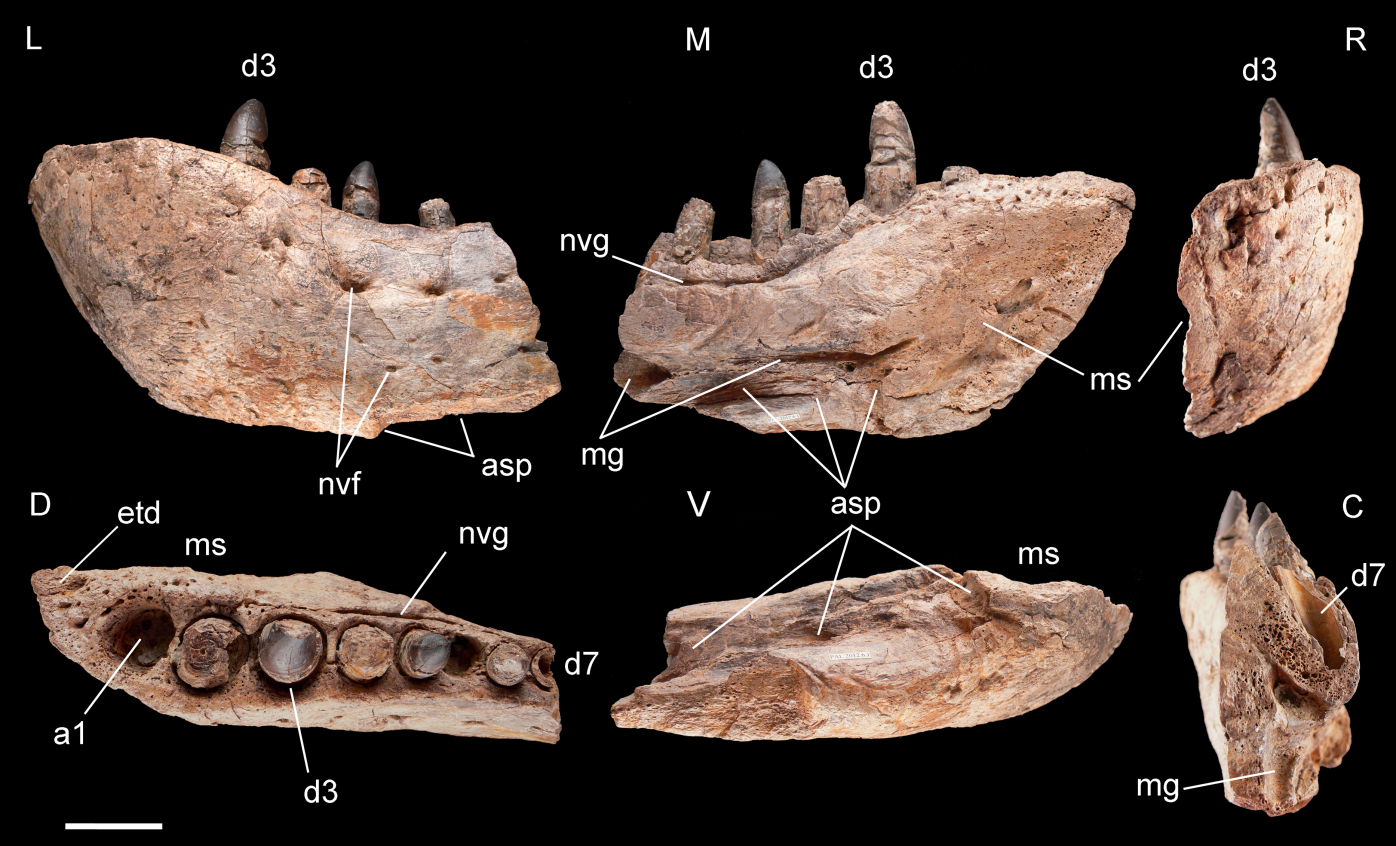
Left dentary of *Razanandrongobe sakalavae.* Specimen MHNT.PAL.2012.6.1 in lateral (L), medial (M) rostral (R), dorsal (D), ventral (V), and caudal (C) views. Scale bar = 5 cm. Abbreviations: see text.

**Figure 5:**
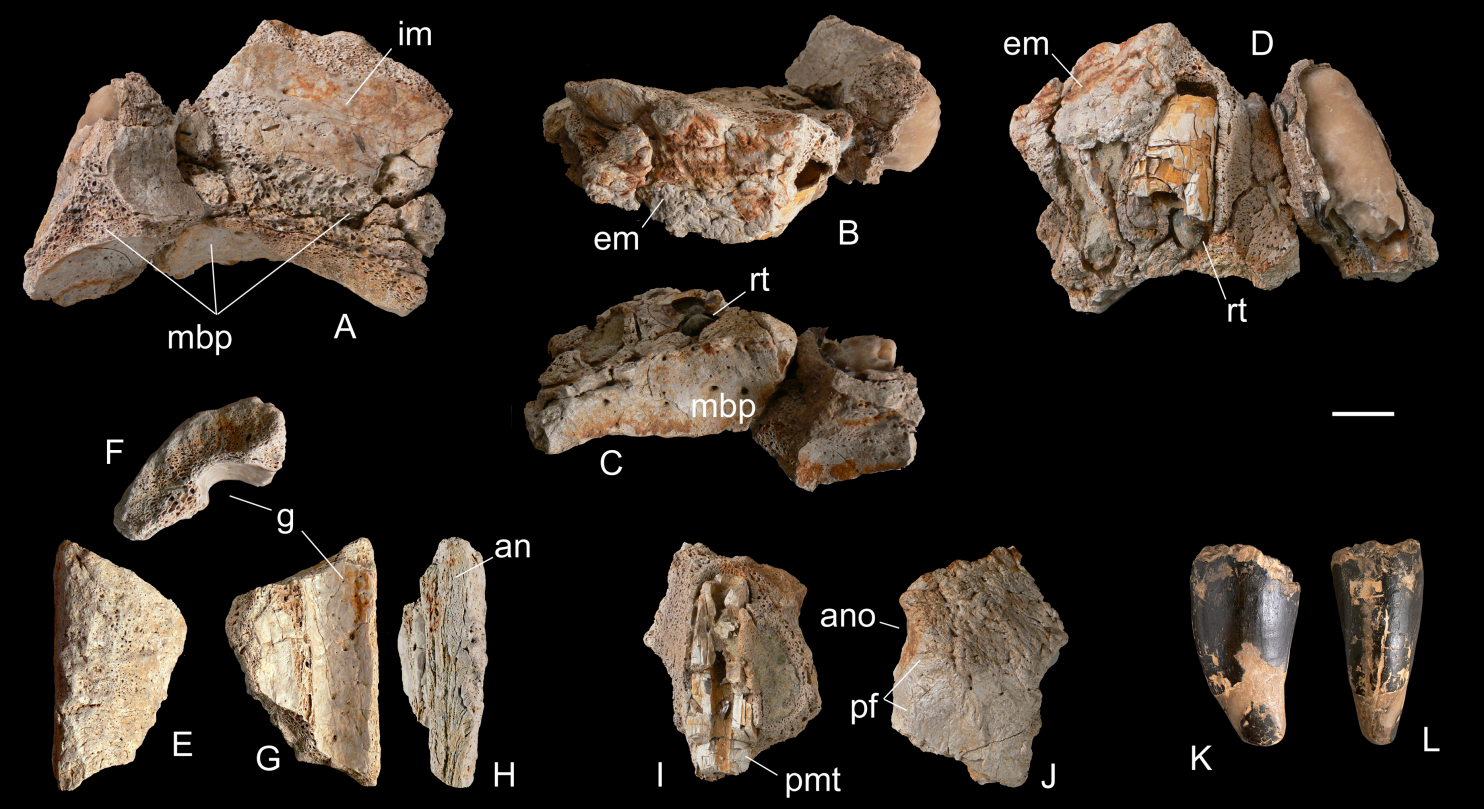
Selected cranial fragments and an isolated tooth referred to *R. sakalavae*. Right maxillary fragment (MHNT.PAL.2012.6.3) in medial (A), dorsal (B), ventral (C), and lateral (D) views; ?left maxillary fragment (MHNT.PAL.2012.6.5) in dorsal (E), caudal (F), ventral (G), and medial (H) views; left premaxillary fragment (MHNT.PAL.2012.6.4) in medial (I) and lateral (J) views; very large isolated tooth (MSNM V7144) in lateral (K) and rostral (L) views. Scale bar = 2 cm. Abbreviations: see text.

**Referred material**—MHNT.PAL 2012.6.2, a complete right premaxilla (Fig. 3); MHNT.PAL 2012.6.1, a left dentary, incomplete caudally (Fig. 4); MHNT.PAL.2012.6.3, a fragmentary right maxillary dentigerous ramus (Figs. 5A–5D); MHNT.PAL.2012.6.4, a premaxillary fragment (Figs. 5I, 5J); MHNT.PAL.2012.6.5, a laterodorsal maxillary fragment (Figs. 5E–5H); MHNT.PAL.2012.6.6–8, three indeterminate skull fragments; MSNM V5771–5777 (Maganuco, Dal Sasso & Pasini, 2006: figs. 7, 8) and V7144 (Figs. 5K, 5L), eight isolated teeth.

**Age and stratigraphic horizon**—Middle Jurassic (Bathonian), 167.7–164.7 MA (Cohen et al., 2013), Mahajanga Basin, Sakaraha Formation (*sensu* Geiger, Clark & Mette, 2004) (Fig. 1).

**Locality**—Hills W of Ambondromamy (maxilla, possibly premaxilla and dentary), hills N-NW of Andranomamy (teeth), and undetermined localities (cranial fragments), Mahajanga Province, NW Madagascar (Fig. 1).

**Emended diagnosis**— The following characters are synapomorphic to *R. sakalavae*: tip of dentary edentulous, for a length surpassing the diameter of the first alveolus; alveoli with labiolingual diameter larger than mesiodistal diameter; mesial teeth incisiform, U-shaped in cross-section, with both carinae facing the lingual side; denticles present on both carinae in all teeth, homogeneous, symmetrically convex, and very large (0.8–1.4 per mm).

**Remarks**—*R. sakalavae* differs from other known crocodyliforms in the following combination of characters: large predatory mesoeucrocodylian with oreinirostral snout; premaxillae taller than long, bearing five teeth and having *aperturae nasi osseae* facing rostrally, confluent medially, and bordered by smooth perinarial fossae; lateral edge of *aperturae nasi osseae* without notch on the premaxilla; very large incisive foramen, with length slightly more than half the greatest width of the premaxilla; premaxillary bony palate (*sensu* Kley et al., 2010) with subcircular paramedian depressions, located rostrally on the premaxilla; paradental shelf of the premaxilla and maxilla with a surface texture consisting of marked ridges and furrows, in the maxilla extending for a short distance also above the medial neurovascular groove; deep robust maxilla, bearing at least ten teeth and a stout maxillary bony palate (*sensu* Kley et al., 2010) located well above the level of the alveolar row; deep robust dentary, bearing at least eight teeth, the largest of which are the first three (fourth hypertrophied caniniform tooth absent); preserved part of the dentary with a convexity followed by a concavity along the dorsal edge; splenial contributing to the mandibular symphysis for at least 20% of the whole symphyseal length; dental implantation in separate alveoli; alveolar channels nearly straight in the sagittal plane; alveoli subrectangular to subcircular in occlusal view, with the former located on the paradental wall of the maxilla and on the rostralmost portion of the dentary; dentition heterodont and pachydont (*sensu* Hendrickx, Mateus & Araújo, 2015); lateral teeth (*sensu* Farlow et al., 1991) stout, suboval to salinon-shaped in cross-section; mid-lateral tooth crowns not compressed, subcircular in cross section; smallest lateral teeth globe-shaped.

## Description

**Premaxilla, MHNT.PAL.2012.6.2** (Fig. 3 and Table 1). The almost complete right premaxilla is taller than long (16 *vs* 13.5 cm), with the premaxillary symphysis straight in rostral and ventral views. This indicates that the rostrum was rostrocaudally short, dorsoventrally deep, mediolaterally wide, and not pointed. The premaxilla bears five teeth that are sub-vertical and only slightly curved lingually.

**Table 1:** Basic numbers and measurements (in mm) of the teeth of the specimens MHNT.PAL.2012.6.2 and MHNT.PAL.2012.6.1. Abbreviations: see text.

Specimen	TCH labial margin	FABL	BW	FABL /BW	FABL/TCH	serr. per 5 mm mesial carina	serr. per 5mm distal carina	DSDI
PAL.2012.6.2 pm1 (repl. tooth)	–	–	–	–	–	5	5	1.00
PAL.2012.6.2 pm2	(41)	(24)	(24)	(1.00)	(0.58)	–	4.5	–
PAL.2012.6.2 pm3	(59)	31	29	1.07	(0.52)	–	–	–
PAL.2012.6.2 pm4	(56)	31	27	1.15	(0.55)	4	4	1.00
PAL.2012.6.2 pm5	(51)	(30)	28	(1.07)	(0.59)	4	–	–
PAL.2012.6.2 pm5 (repl. tooth)	–	–	–	–	–	4	–	–
PAL.2012.6.1 d1	–	–	–	–	–	–	–	–
PAL.2012.6.1 d2	–	[28]	[27]	[1.04]	–	–	–	–
PAL.2012.6.1 d3	(48)	27	28	0.96	(0.56)	5	4.5	1.11
PAL.2012.6.1 d4	(23)	(27)	(26)	(1.04)	(1.17)	–	–	–
PAL.2012.6.1 d5	16	(20)	(21)	(0.95)	(1.25)	6	5.5	1.09
PAL.2012.6.1 d6 (repl. tooth)	–	–	–	–	–	5	5	1.00
PAL.2012.6.1 d7	(23)	18	16	1.12	(0.78)	–	–	–
PAL.2012.6.1 d8	–	[19]	[19]	[1.00]	–	–	–	–
MSNM V7144 Isolated ?m tooth	(67)	35	31	1.13	(0.52)	–	–	–

**Notes.**

() preserved.

[] estimated.

In medial and ventral views, two subcircular paramedian depressions are visible, corresponding to the tip of the mesialmost dentary tooth crowns at jaws closed. The bone wall at one depression is damaged, revealing a well-developed replacement tooth. Dorsal to these depressions is the surface for contact with the palatal portion of the maxilla, namely the rostral portion of the maxillary bony palate. Rostrally, this palatal shelf did not reach the symphysis between the two premaxillae, but instead left an open space in the palate (the incisive foramen) bordered rostrally and laterally by the premaxillae. A subtriangular notch for the premaxillary peg of the maxilla is visible dorsal to the depression hosting the dentary teeth. The rest of the premaxillomaxillary suture is almost flat. There is no lateral groove in the premaxilla for reception of a hypertrophied lower caniniform tooth.

The *apertura nasi ossea* (often improperly called the external naris, which instead refers to the fleshy nostril) is bordered caudolaterally and ventrally by the premaxilla, and dorsally—in all likelihood—by the nasal, whereas rostromedially it clearly lacks any trace of a dorsally directed nasal process along the medial sagittal plane. Therefore, the left and right *aperturae nasi osseae* did not only face rostrally, but were also confluent medially.

The surface of the perinarial fossa is smooth and lateromedially wide, and extends from the caudoventral margin of the *apertura nasi ossea* to the alveolar margins of premaxillary teeth 1–3 ventrally, and to the ornamented facial portion of the premaxilla caudally. The premaxilla is also dorsolaterally rough and ornamented with small crests, pits, and fine grooves. Near the alveolar margin, the ornamented palatal surface, visible in medial view, is pitted by small neurovascular foramina, which are more abundant in a groove delimiting the dorsal end of the paradental shelf. Dorsally, the walls of the premaxilla taper up to form an extended (80 mm long) attachment area for the nasal.

The premaxilla bears four erupted teeth implanted in alveolar positions 2–5. The tooth replacement process is described in detail for the holotype maxilla in Maganuco, Dal Sasso & Pasini (2006). However, CT of the new specimen has provided the following additional information on this process. Resorption of the roots is apparent at positions 2 and 5, in correspondence with the growing replacement teeth, but the roots are still firmly developed and the replacement teeth are growing mostly medial/mesiomedial to the roots. The tips of the replacement teeth are 36 mm from the ventral surface of the premaxilla. At position 3, resorption of the root is more advanced: the tip of the replacement tooth is 22 mm away from the ventral surface of the premaxilla, and is aligned with the erupted tooth. The first alveolus is occupied by a large unerupted replacement tooth. The limited depth of the tooth-bearing portion of the premaxilla in correspondence with the first alveolus (i.e., below the *apertura nasi ossea*) and the diameter of the alveolus itself indicate that the first premaxillary tooth, even when completely grown, was less than 80% the height of the other premaxillary teeth. Moreover, CT scanning revealed that the roots are straighter mesially than distally when seen in lateral view.

**Dentary, MHNT.PAL.2012.6.1** (Fig. 4 and Table 1). The left dentary is incomplete caudally. In lateral view, its maximum dorsoventral depth/height (13 cm) is at the level of alveolus 3. The bone then tapers caudally up to the level of alveolus 6, where the dorsal and ventral margins become almost parallel. There is no constriction at or near the mandibular symphysis along the lateral margin. The rostral edge of the symphysis forms an angle of about 50° with the ventral margin of the dentary. The preserved portion of the dentary bears eight large mandibular teeth; however, none can be considered a hypertrophied caniniform tooth. The size of the alveoli varies along the tooth row. Based on alveolar diameter, the mesial teeth are the largest, numbers 4 and 5 slightly decreasing in size; from 6 onward, their size is constantly half the diameter of alveoli 2 and 3. The position of the first alveolus indicates that the tip of the lower jaw is edentulous. Of the eight tooth positions, number 1 is represented by an empty alveolus, number 6 by a replacement tooth erupting from its alveolus, number 7 by a longitudinally broken and empty crown, numbers 2, 4, and 6 by broken crowns without apex, and only numbers 3 and 5 by almost complete crowns.

The mandibular symphysis with the counterlateral element is 16 cm long and extends caudally to the level of the third tooth, but considering also the estimated contribution of the splenial, it probably extended posteriorly to the level of the fourth tooth. The splenial itself is not preserved, and is represented by a scar; its apex terminated rostrally in a squared peg at the level of the third tooth, as indicated by rostrocaudally elongate sutural marks on the dentary; it contributed to at least 20% of the length of the mandibular symphysis. Caudal to the symphysis, the splenial formed the medioventral portion of the mandibular ramus, resulting visible also in lateral view. The Meckelian groove is exposed in medial view: rostrally, it reaches the mandibular symphysis; caudally, it fades into the contact surface for the splenial.

The lateral surface of the dentary is as well-ornamented as the lateral surface of the premaxilla, and is densely sulcated by rostrocaudally oriented zigzagging vascular canals. Dorsomedially, the bone bears one row of neurovascular foramina parallel to the alveolar margins; internal nutritive foramina bordering alveoli 1–3; and foramina caudal to alveolus 3 aligned in a grooved row closely flanking the lingual margin of the remaining alveoli.

**Maxillary fragment,** **MHNT.PAL.2012.6.3** (Figs. 5A–5D). This incomplete maxillary ramus has six incomplete, straight to slightly curved alveoli, as well as two tooth roots and at least one unerupted tooth. The curvature of the bone, the shape of the roots in lateral and medial views, and the position of the replacement tooth with respect to the alveoli indicate that this specimen is part of the right side of the skull. Moreover, the increasing curvature of the rostral portion of the specimen is suggestive of close proximity to the premaxillary contact, permitting us to designate the alveoli as belonging to teeth 1–6 or 2–7. Of note, the interior of the mesialmost tooth has been replaced by a cast made from a coarse-grained, pale-brownish calcite nodule of alabastrine appearance (F Pezzotta, pers. comm., 2016). Position 4 (or 5) still houses the fossilized root dentine of a slightly larger tooth and, mesiomedially, the tip of a replacement tooth protruding vertically down and exposing the large denticles that are diagnostic of *R. sakalavae*. A small portion of the lateral (external) maxillary wall is preserved caudodorsally to this tooth position, showing the rugose texture seen in the caudal portion of the premaxilla MHNT.PAL.2012.6.2. In rostral view, a triradiate-shaped broken section of the bone marks the remains of the maxillary bony palate, at mid-height of the preserved alveolar height. Dorsal to the shelf, the medial (internal) wall of the maxilla shows a wide concave surface, relatively smooth where the bone is not broken; ventral to the shelf, the bone of the palate is even smoother, with larger nutritive foramina, but is much more concave (this is the transition point from the palate to the dentigerous margin).

**Premaxillary fragment,** **MHNT.PAL.2012.6.4** (Figs. 5I–5J). This is a small fragment of bone with three half-sections of alveoli, the middle one still housing a tooth root, broken longitudinally and with the pulp cavity exposed. Orienting the alveoli vertically, the external wall can be seen divided into a rugose dorsal portion and a smoother ventral portion; the latter continues caudodorsally, curving inward into a concave area. By comparison with specimen MHNT.PAL.2012.6.3, we interpret this concave area as a caudolateral tract of the *apertura nasi ossea*, and the smooth ventral portion as part of the perinarial fossa. Therefore, this bone fragment likely comes from a left premaxilla.

**Maxillary** **fragment,** **MHNT.PAL.2012.6.5** (Figs. 5E–5H). This is a trapezoidal fragment of ornamented bone. The internal wall is traversed by a large, smooth, concave groove. Parallel to one margin of the groove is a long, exposed longitudinal suture. We tentatively refer this fragment to a mediodorsal portion of the ?left maxilla likely contacting the nasal via the aforementioned suture. If the fragment has been correctly placed, the ornamentationwould represent transverse ridges and the groove might be part of a pneumatic space of the maxilla, parallel to the internal choanae, which in mesoeucrocodylians run more medially, below the nasals (e.g., *Alligator,* Brochu, 1999).

**Indeterminate fragments,** **MHNT.PAL.2012.6.6–8.** These three specimens are so fragmentary that it is impossible to determine their anatomical positions. However, we tentatively propose cranial or mandibular origins for MHNT.PAL.2012.6.6 and MHNT.PAL.2012.6.8, for the former because of the presence of a heavily rugose and sulcate ?external surface that fades to a smooth texture ?internally via a convex, U-shaped margin, and for the latter because it has a zigzagging suture mark and a possible internal concavity somewhat reminiscent of a cranial opening. MHNT.PAL.2012.6.7 remains totally indeterminate, and is mentioned here only because it is associated with the other fragments.

**New remarks on the holotype maxilla, MSNM V5770** (Maganuco, Dal Sasso & Pasini, 2006: figs. 3–7). The holotypic cranial fragment of *R. sakalavae* consists of a portion of right maxilla bearing a markedly rugose paradental shelf, a robust sub-horizontal maxillary bony palate positioned definitely higher (dorsal) to the row of alveoli, five large subvertical alveoli, and three unerupted teeth that illuminate the process of tooth replacement (see Maganuco, Dal Sasso & Pasini, 2006 for details). Thanks to the new material described here, the phylogenetic affinities of *R. sakalavae* have become clearer. Moreover, they have shed light on some previously uncertain features of the holotype maxilla, such as its position in the skull, some doubtful attachment areas (palatine or ectopterygoid; jugal or lacrimal), the presence/absence of the antorbital fenestra, and the margin of the suborbital fenestra. MSNM V5770 bears the caudal portion of the maxillary tooth row, as indicated by the tapering of the paradental shelf, the ending of the maxillary bony palate, and by the scar on the medial surface of the caudalmost portion of the bone that represents the attachment area for the ectopterygoid. Therefore, the rostralmost portion of the ectopterygoid is rostral to the distal alveoli. The thickest part of the maxillary bony palate is confirmed to be the attachment area for the palatine (Kley et al., 2010: figs. 7D, 7E), as previously suggested by Maganuco, Dal Sasso & Pasini (2006), and participates to the rostral margin of the suborbital fenestra. These features render the palate of *Razanandrongobe sakalavae* quite basal-ziphosuchian-like [e.g., *Araripesuchus* (Pol & Apesteguía, 2005)], and not at all baurusuchian-like [e.g., *Pissarrachampsa* (Montefeltro, Larsson & Langer, 2011)] or peirosaurid-like [e.g., *Hamadasuchus* (Larsson & Sues, 2007)]. It is impossible to estimate how long the missing rostral portion of the maxilla was, but we have at least some indication of the minimum length thanks to the new specimens (see, Cranial reconstruction and size). The caudolateral surface visible in dorsal view most likely represents the remaining portion of the attachment area for the jugal (e.g., Larsson & Sues, 2007: fig. 2) rather than the pavement of the antorbital fenestra.

### Dentition

The dentition of *R. sakalavae* consists of five premaxillary and at least ten maxillary and eight dentary teeth. In occlusal view, the medial halves of the alveoli in the holotype maxilla and in the rostralmost portion of the dentary are subrectangular, whereas all other alveolar margins are subcircular. In complete alveoli, the labiolingual width exceeds the mesiodistal length (Table 2). Based on the alveolar diameters, the premaxilla and dentary belong to an individual comparable in size to that of the holotype.

**Table 2:** Measurements (in mm) of the alveoli of the specimens MHNT.PAL.2012.6.2 and MHNT.PAL.2012.6.1. Abbreviations: see text.

Specimen	Alveolar maximum (labiolingual) diameter	Alveolar minimum (mesiodistal) diameter
PAL.2012.6.2 pm1 (repl. tooth)	32	20
PAL.2012.6.2 pm2	32	25
PAL.2012.6.2 pm3	36	29
PAL.2012.6.2 pm4	40	33
PAL.2012.6.2 pm5	35	[30]
PAL.2012.6.1 d1	32	28
PAL.2012.6.1 d2	36	31
PAL.2012.6.1 d3	37	33
PAL.2012.6.1 d4	31	27
PAL.2012.6.1 d5	28	24
PAL.2012.6.1 d6 (repl. tooth)	20	19
PAL.2012.6.1 d7	20	20
PAL.2012.6.1 d8	[19]	17

**Notes.**

[] estimated.

The alveolar implantation and morphology in the new specimens is as described by Maganuco, Dal Sasso & Pasini (2006) for the maxilla. The teeth are closely spaced, separated only by thin interdental walls that are proportionally thicker between the smaller dentary teeth and thinner between the largest premaxillary teeth. Additional features seen with CT are the roots, straight mesially and slightly procurved distally in lateral and medial views, and the replacement teeth, erupting medially.

**Figure 6:**
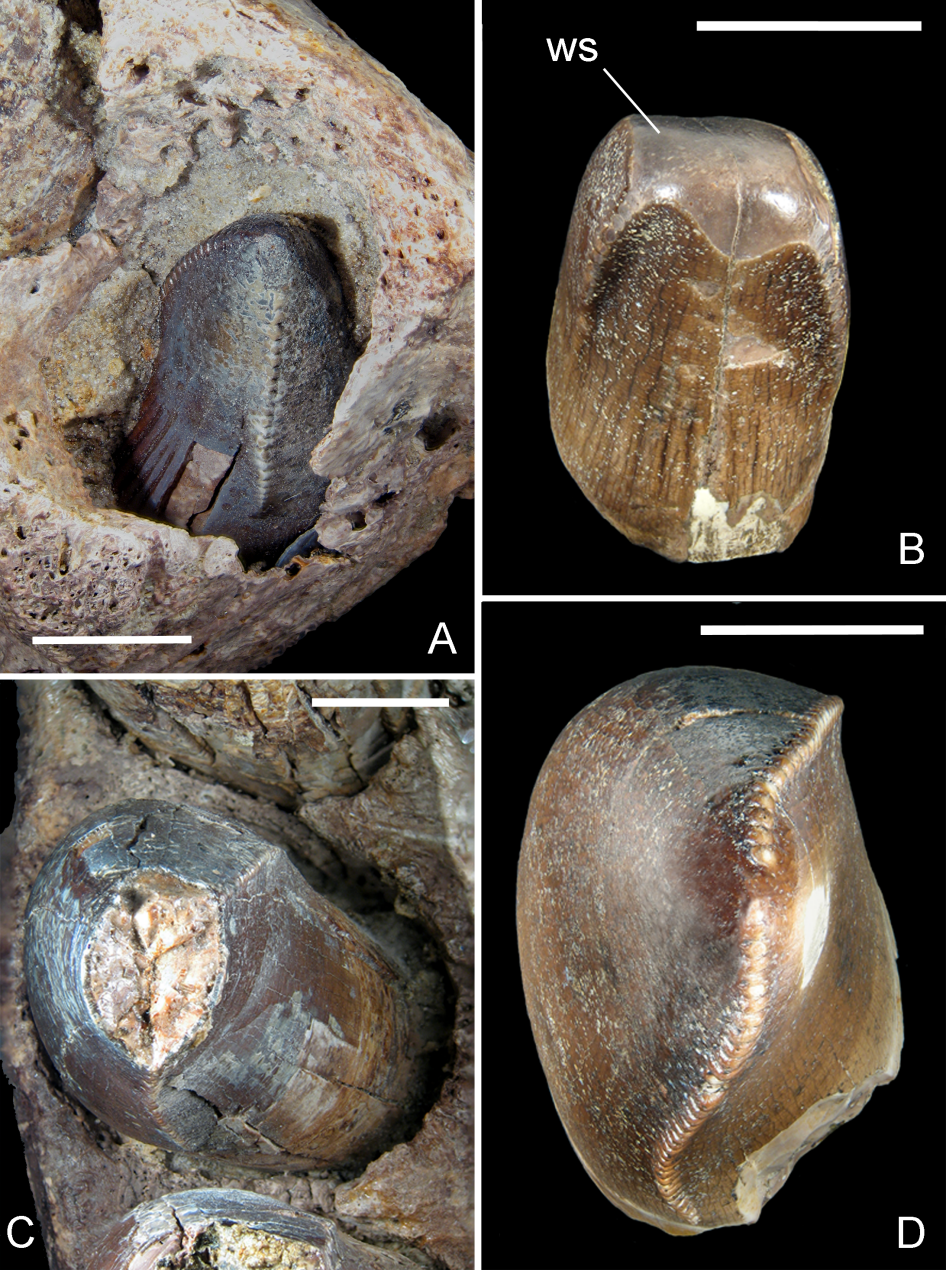
Mesial and lateral teeth of *R. sakalavae.* (A) replacement tooth pm1 in MHNT.PAL.2012.6.2; (B) mesial functional tooth MSNM V5775; (C) pm4 in MHNT.PAL.2012.6.2; (D) lateral tooth of indeterminate position MSNM V5773. Scale bars = 10 mm. Abbreviations: see text.

The teeth of *R. sakalavae* were thoroughly described by Maganuco, Dal Sasso & Pasini (2006, and references therein), so only a summary of their features is presented here. The crowns are serrated with well-defined, regularly spaced denticles that are remarkably large [i.e., even larger than the largest denticles in large-bodied theropods, *Tyrannosaurus rex* included (Brochu, 2003)]; at mid-crown, there are 0.8–1.4 denticles per mm. The mesial teeth are U-shaped in cross section, with both carinae on the lingual side. The denticles are slightly inclined toward the tip of the crown. The lateral teeth are strongly asymmetrical, with a marked twist of the mesial carina: in cross section, they appear to be salinon-shaped (*sensu* Hendrickx, Mateus & Araújo, 2015) at mid-crown, and suboval at the crown base, with a labiolingual diameter often larger than the mesiodistal one. Thus, the teeth of *R. sakalavae* are labiolingually more inflated than usually found in ziphodont crocodylomorphs and in tyrannosaurid theropods (e.g., Prasad & de Lapparent de Broin, 2002; Brochu, 2003). The tips of most teeth are markedly worn, with spalled surfaces extending to the lingual and labial faces and broadly exposing dentine with microscopic wear striations (Fig. 6B). Most of the enamel surface is missing from the majority of crowns.

The best preserved teeth (i.e., the fourth premaxillary and the fifth dentary) confirm that there is no significant basal constriction between the crown and the root, the latter being only slightly narrower. The mesialmost teeth of the premaxilla are either only slightly smaller than or as large as the lateral ones; the mesial dentary teeth are even larger than the teeth distal to the symphysis. Therefore, the mesial tooth crown MSNM V5775 (Maganuco, Dal Sasso & Pasini, 2006)— which is U-shaped in cross section and matches the shape of the erupting tooth in the first premaxillary alveolus in specimen MHNT.PAL.2012.6.2 (Figs. 6A, 6B)—clearly came from an individual that was almost half the size of the one from which MHNT.PAL.2012.6.2 belonged.

CT has provided measurements of the tooth crown and root: the largest dentary and premaxillary teeth (d3 and pm3) are 13.76 and 15.04 cm long, respectively. The root of the latter fully encloses a 6.41-cm-long replacement tooth.

**MSNM V7144** (Figs. 5K, 5L) is the largest tooth of *R. sakalavae* so far recorded. Indeed, its basal width (Table 1) is larger than that of the third premaxillary tooth of MHNT.PAL.2012.6.2 by 2 mm. It lacks the apical third of its enamel and appears to have had the tip of the crown worn out (and possibly further eroded by postdiagenetic weathering). The only remnant of distal carina is preserved without any complete denticle, although four denticle bases, each 1 mm in diameter, are faintly visible at the base. Besides denticle size, referral to *R. sakalavae* is based on tooth crown shape and robustness and on the subcircular cross-section of the crown base. The size of this isolated crown fits well with a tooth at (relative) position 3 of the holotype maxilla, as indicated also by comparisons made with the implanted teeth in MHNT.PAL.2012.6.1 and MHNT.PAL.2012.6.2 and with the sizes of the alveoli in MSNM V5770. Its cross section, which is symmetrical and not U- or D-shaped, and its large size are also indicative of a maxillary tooth.

Many dental features, coupled with the massive nature of the jaw bones, suggest that the carnivorous diet of *R. sakalavae* included bone and tendon. Indeed, the worn surfaces on *R. sakalavae* teeth (Maganuco, Dal Sasso & Pasini, 2006: figs. 8H, 8I) appear more like areas of ante-mortem enamel spalling caused by contact with food rather than attritional facets caused by tooth–tooth contact (Schubert & Ungar, 2005). It is possible that flakes of enamel were splayed off the underlying dentine surfaces as the animal bit into bones or other hard objects (Currie, Rigby Jr & Sloan, 1990). Similar wear facets can be seen in an average of 1/10 isolated theropod teeth found at Dinosaur Provincial Park (D Tanke & P Currie, pers. comm., 2004). Among the erupted teeth of *R. sakalavae* that were not damaged post-mortem, five out of seven present such a spalled surface (Maganuco, Dal Sasso & Pasini, 2006: fig. 7). In addition, the mesial incisiform tooth MSNM V5775 (Fig. 6B) closely resembles tyrannosaurid premaxillary teeth, which were more likely used in scraping meat from bone than in capturing prey (Erickson, 1999). Finally, the denticles of *R. sakalavae* appear very similar to those of tyrannosaurids in size, shape, basal width, and presence of a round void at the base of the junction between neighboring denticles. Such a morphology seems to be typical of strengthened denticles well adapted for biting into bone (Abler, 2001; Currie, Rigby Jr & Sloan, 1990).

## Discussion and Comparisons

### Why *Razanandrongobe sakalavae* cannot be a theropod

The new *R. sakalavae* material displays the following key features that are unequivocally crocodylomorph and that do not match the anatomy of theropod dinosaurs in any way: (1) *aperturae nasi osseae* facing rostrally, broadly excluded from rostrolateral exposure and from maxillary contact, and clearly confluent, without any premaxillary nasal process directed dorsally and dividing them along the medial sagittal plane; (2) mandibular symphysis more robust and more elongate rostrocaudally than in any theropod; (3) splenial integrated in the mandibular symphysis, as in all Mesoeucrocodylia, and forming a conspicuous ventromedial portion of the lower jaw, resulting visible also in lateral view (e.g., Schwarz, 2002; Pol & Apesteguía, 2005; Schwarz & Salisbury, 2005; Carvalho, Vasconcellos & Tavares, 2007; Martinelli et al., 2012); (4) bony palate well-developed on the maxilla as in many crocodylomorphs (no theropod taxon has a maxillary bony palate as well developed and stout as in *R. sakalavae*); and (5) alveoli and tooth crowns labiolingually expanded and inflated, a feature completely different to that seen in theropods, including those reported from Madagascar (abelisaurids), which have laterally compressed tooth crowns. In actual fact, no theropod group apart from the Spinosauridae has large and labiolingually thick conical teeth expanded to the same degree, whereas conical crowns are common in crocodylomorphs (e.g., Prasad & de Lapparent de Broin, 2002); however, spinosaurid theropods differ greatly from *Razanandrongobe* in many cranial features, such as the retracted position of the *apertura nasi ossea* (e.g., Sereno et al., 1998; Dal Sasso et al., 2005).

Because these key features were not discernible in the holotype of *R. sakalavae*, Maganuco, Dal Sasso & Pasini (2006) listed and commented on similarities with other archosaurian taxa, including theropod dinosaurs, that now result to be convergent or non-homologous features.

### Comparisons within Crocodylomorpha

The new material confirms the conclusion in Maganuco, Dal Sasso & Pasini (2006) that the taxon is clearly different from the basal crocodylomorphs. In particular, it differs from the “sphenosuchians” (Wu & Chatterjee, 1993; Sues et al., 1996; Clark, Sues & Berman, 2000) mainly in a number of dental characters, including: (1) the absence of a basal tooth crown constriction; (2) the presence of very large, well-defined denticles, also on mesial teeth; (3) lateral teeth not labiolingually compressed; and (4) a dentary bone deeper and more robust than in the “sphenosuchian grade” *Macelognathus* (Göhlich et al., 2005), which also has a much longer edentulous rostral portion and, above all, a very limited, if any, contribution of the splenial to the mandibular symphysis. A small symphyseal area with no participation of the splenial is reported for the sphenosuchian *Terrestrisuchus* (Crush, 1984). In addition, Maganuco, Dal Sasso & Pasini (2006) already pointed out that the palate of *R. sakalavae*, when reconstructed on a sphenosuchian blueprint, would differ in having a more developed maxillary bony palate, more retracted internal choanae, and antorbital fenestra smaller rostrally and more caudally displaced. Following our re-interpretation of the anatomy of the holotype maxilla given above (see New remarks on the holotype maxilla, MSNM V5770), features like the position of the attachment area of the ectopterygoid and the participation of the maxilla to the suborbital fenestra clearly indicate that the bone topology of *R. sakalavae* does not exactly match that of the sphenosuchians, but rather that it can be compared with that of the mesoeucrocodylians.

*Razanandrongobe sakalavae* had ***aperturae nasi osseae*** (which correspond with the external nares) directed rostrally. This orientation is different to that of the Mahajangasuchidae (Turner & Buckley, 2008), in which they opened dorsally, but similar to that of the Uruguaysuchidae (Soto, Pol & Perea, 2011) and most non-neosuchian crocodyliforms (e.g., Pol et al., 2014). In the Peirosauridae (*Montealtosuchus; Hamadasuchus*), the external nares faced laterally as well as somewhat rostrally, and were separated by a robust bony bar (Carvalho, Vasconcellos & Tavares, 2007; Larsson & Sues, 2007). However, the *aperturae nasi osseae* in *Razanandrongobe* are confluent, as the premaxillae do not contribute to any septum and there are no traces of an attachment for a descending nasal septum. The external nares faced rostrally also in the Baurusuchidae (*Aplestosuchu*; *Gondwanasuchus*; *Stratiosuchus*), but were divided by a septum formed ventrally by the premaxilla and dorsally by the nasal, which projects rostrally beyond the alveolar margin of the premaxilla (Carvalho, Ribeiro & Avilla, 2004; Campos et al., 2001; Godoy et al., 2014; Marinho et al., 2013). The *aperturae nasi osseae* are completely septate also in the Sebecidae (*Lorosuchus*) but, differently to *R. sakalavae*, the nares faced dorsally (Pol & Powell, 2011). Although highly derived in several anatomical characters of the skull and lower jaw, the Sphagesauridae (Pol et al., 2014) share with *R. sakalavae* external nares that were confluent, rostrally positioned, and bordered by the same bones [*contra* the marine taxon *Dakosaurus*, in which the nasal does not reach the premaxilla and does not participate to the *apertura nasi ossea* (Gasparini, Pol & Spalletti, 2006)].

*Razanandrongobe* has a distinct **perinarial fossa**. According to Martinelli et al. (2012), this fossa is widely distributed among mesoeucrocodylians, from Peirosauridae (*Uberabasuchus*; *Montealtosuchus*; *Hamadasuchus*; *Peirosaurus*; *Gasparinisuchus*) to Uruguaysuchidae (e.g., *Araripesuchus*), and from *Simosuchus* to sebecosuchians (*Iberosuchus*; *Baurusuchus*; *Stratiosuchus*; *Wargosuchus*). Therefore, this is likely plesiomorphic for the Notosuchia.

The premaxilla of *Razanandrongobe* lacks a **paracanine notch** or fossa, which some mesoeucrocodylians and some notosuchians [Peirosauridae and Sebecosuchia (Baurusuchidae; Sebecidae)] have for reception of an enlarged dentary caniniform tooth (also absent in *R. sakalavae*). The condition seen in *Razanandrongobe* is comparable to that of some crocodylomorphs, such as the Jurassic *Hsisosuchus*, and basal Ziphosuchia, like *Candidodon*, *Malawisuchus*, *Simosuchus*, *Pakasuchus*, and *Libycosuchus* (Fiorelli, pers. comm., 2016). However, also several advanced notosuchians, such as *Notosuchus* and *Mariliasuchus*, do not have this notch (Pol et al., 2014). According to L Fiorelli (pers. comm., 2016), the lack of a paracanine notch would be plesiomorphic to Notosuchia, as so far it has only been encountered in Peirosauridae and Sebecosuchia.

There is a very large **incisive foramen** in *Razanandrongobe*; the length of this foramen is equal to, or more than half, the premaxillary width. Several Mesoeucrocodylia, including sphagesaurids, peirosaurids, baurusuchids, and neosuchians, show foramina of smaller size (e.g., Larsson & Sues, 2007; Pol et al., 2014). In Sebecidae (*Lorosuchus*), the palatal branches of the premaxillae enclose a subovoid incisive foramen, which again is definitely smaller than in *Razanandrongobe*.

The **maxillary bony palate** of *Razanandrongobe* is robust and extends medially, where it likely formed the extensive secondary palate characteristic of Mesoeucrocodylia. In Uruguaysuchidae (*Uruguaysuchus*), for instance, the palatal processes of the maxillae are well-preserved, broad, and sutured to one another and to the palatines (Soto, Pol & Perea, 2011). A massive, typically oreinirostral, maxilla is also present in the Sphagesauridae (*Sphagesaurus*; *Adamantinasuchus*; *Armadillosuchus*) and the Baurusuchidae. In particular, the deep maxilla of *Stratiosuchus maxhechti* (Campos et al., 2001), with its convex ventral margin bearing five teeth, is reminiscent of the holotype of *R. sakalavae*. In Mahajangasuchidae (*Mahajangasuchus*), the maxilla is extremely low in lateral profile, and in Peirosauridae (*Hamadasuchus*; *Montealtosuchus*) it is moderately deep.

In *Razanandrongobe*, the lateral surface of the **dentary**, along the mid-lateral tooth row, is oriented vertically below the alveolar margin, which is continuous with the rest of the lateral surface of the dentary. This character is seen also in Uruguaysuchidae (*Araripesuchus*), Mahajangasuchidae, Peirosauridae, and in derived notosuchians (Fiorelli et al., 2016), including baurusuchids [e.g., *Gondwanasuchus* (Marinho et al., 2013)] and sebecids [e.g., *Sahitysuchus* (Kellner, Pinheiro & Campos, 2013)]. In lateral view, the ventral margin of the deep dentary of *Razanandrongobe* extends horizontally up to the level of the third dentary tooth. Rostral to this point, the dentary turns upward in a rostrodorsal extension, at an angle of about 50° with the long axis of the bone, giving the tip of the dentary the aspect of a very strong bone. This structural character, which probably evolved to allow increased biting forces, is very much like that described for the Baurusuchidae (*Aplestosuchus*; *Gondwanasuchus*; *Pisarrachampsa*)—including the angle, which is approximately 45°— (Godoy et al., 2014; Marinho et al., 2013; Montefeltro, Larsson & Langer, 2011) and the mahajangasuchid *Kaprosuchus* (Sereno & Larsson, 2009). Somewhat different and lighter constructions are seen in Uruguaysuchidae (*Uruguaysuchus*) and Peirosauridae (*Hamadasuchus*; *Monteoaltosuchus*; *Pepesuchus*), in which the dentary tapers rostrally in a continuous upward arch (Larsson & Sues, 2007; Carvalho, Vasconcellos & Tavares, 2007; Campos et al., 2011), and in the mahajangasuchid *Mahajangasuchus*, in which the tip of the mandible is not as deep rostrally as in *R. sakalavae* (Turner & Buckley, 2008).

From the splenial suture preserved on the dentary of *R. sakalavae,* we infer that the **splenial** contributed to a least 20% of the mandibular symphysis. This is an important character, but we cannot provide a precise percentage due to the lack of the splenial itself. The value is close to 20% in advanced notosuchians (e.g., *Notosuchus*, *Caipirasuchus*), as well as in *Comahuesuchus* and sebecosuchians [e.g., *Gondwanasuchus* (Marinho et al., 2013) and *Sahitysuchus* (Kellner, Pinheiro & Campos, 2013)]. In contrast, it is close to 30% in basal ziphosuchians (e.g., *Candidodon, Malawisuchus*, *Simosuchus*, *Pakasuchus*) and in uruguaysuchids (Gomani, 1997; Sereno & Larsson, 2009; O’Connor et al., 2010; Soto, Pol & Perea, 2011). It is obvious that the condition seen in our taxon does not match that of the Peirosauridae (*Hamadasuchus*; *Monteoaltosuchus*; *Pepesuchus*), in which the symphysis extends to the 10th/11th dentary tooth: indeed, such a condition in *Razanandrongobe* would imply a contribution of the splenial to the symphysis equal to 50%.

Again, from the sutural marks on the dentary, we infer that, contrary to *Araripesuchus* (Pol & Apesteguía, 2005) and *Simosuchus* (Kley et al., 2010), caudal to the mandibular symphysis the splenial of *Razanandrongobe* did not expand dorsally up to the alveolar margin to cover the entire medial surface of the mandibular rami. Instead, the conjoined splenials formed nearly the entire length of the strictly ventral surface of the symphysis, and rostral to that, the rostrodorsally directed symphysis was formed solely by the dentaries. This is identical to the condition seen in the baurusuchid *Pisarrachampsa* (Montefeltro, Larsson & Langer, 2011).

The **alveoli** in *Razanandrongobe* are oriented vertically, as in some terrestrial crocodylomorphs (e.g., “sphenosuchians”) and in several notosuchian lineages (e.g., uruguaysuchids, peirosaurids, baurusuchids, sebecids). *R sakalavae* certainly differs from the Uruguaysuchidae (*Uruguaysuchus*), which are characterized by alveoli in the premaxilla and dentary that lack complete interalveolar septa and, thus, that are all confluent with one another, as well as from the baurusuchid *Aplestosuchus* and the sebecid *Sebecus*, which possess the first alveolus directed forward and, consequently, have a procumbent first dentary tooth. On the other hand, *Razanandrongobe* resembles some Peirosauridae (Martinelli et al., 2012) in having the maxillary and dentary alveoli subrectangular, with planar lingual faces and convex labial surfaces.

Among Mesoeucrocodylia, the **dentition** of *Razanandrongobe* results moderately heterodont, with a mesio-lateral variation that is relatively common except in the incisiform mesial teeth, which are U-shaped in cross-section. The teeth of *R. sakalavae* are not greatly reduced in number, very strongly differentiated in size, or laterally compressed, in contrast with the Baurusuchidae (*Aplestosuchus*; *Baurusuchus*; *Gondwanasuchus*; *Pisarrachampsa*). *Monteoaltosuchus* and *Pepesuchus* (Peirosauridae) show some similarity in the number of premaxillary teeth (i.e., five) that progressively increase in size posteriorly (in *Razanandrongobe*, the largest teeth are the third and fourth), but they are not U-shaped: indeed, all premaxillary crowns are conical. In addition, the teeth of the premaxilla (except for the first), maxilla, and dentary in *Monteoaltosuchus* bear a constriction between the crown and root (a feature absent in our taxon) and fine serrations. The same occurs in *Aplestosuchus* (Baurusuchidae) and *Sebecus* (Sebecidae), in which the premaxillary and mesial dentary teeth are rounded in cross section and all teeth have a basal constriction.

The subcircular to suboval mid-lateral teeth of *R. sakalavae* certainly differ from those seen in most advanced Notosuchia, and are likely a plesiomorphic trait or a feeding adaptation. As a matter of fact, with the exception of the Sphagesauridae, which evolved peculiar subconical teeth (Pol et al., 2014), and the Uruguaysuchidae (*Uruguaysuchus*), in which the dentition is characterized by ‘postcaniniform’ spatulated teeth with strong labiolingual compression, a pointed central cusp, and minute denticles, in Mahajangasuchidae, Peirosauridae, Baurusuchidae, and Sebecidae the mid-lateral teeth are always laterally compressed.

The dentition of *Razanandrongobe* is definitely pachydont [*sensu* Hendrickx, Mateus & Araújo (2015)]: the teeth are labiolingually expanded, incrassate, non-constricted, and have a distally recurved crown in which labiolingual width is greater than 60% of mesiodistal length. The presence of apparently serrated carinae is a distinct feature that was often considered as typical of a ziphodont dentition (e.g., Prasad & de Lapparent de Broin, 2002; De Andrade et al., 2010). However, the definitions given of ziphodont [including one recently improved on by Hendrickx, Mateus & Araújo (2015)] indicate a tooth that is not only serrated but also labiolingually compressed (with labiolingual width less than 60% of mesiodistal length). This is not the case in *R. sakalavae*. Typically ziphodont, for example, are the maxillary teeth and the non-symphyseal dentary teeth of the Baurusuchidae (*Aplestosuchus*; *Gondwanasuchus*; *Pisarrachampsa*) and Sebecidae (*Sahitysuchus*). Pachydont teeth are characteristic of the Tyrannosauridae, but are also present in some notosuchians, such as *Notosuchus terrestris* (Lecuona & Pol, 2008) and *Hamadasuchus* (Larsson & Sues, 2007).

The tooth **denticles** of *Razanandrongobe* are larger than in any mesoeucrocodylian. They are considerably larger than the fine denticles of the other Ziphosuchia (Prasad & de Lapparent de Broin, 2002), and contrast strikingly with the finely denticulate carinae of peirosaurids [e.g., *Gasparinisuchus* (Martinelli et al., 2012)]. In addition, ziphodont mesoeucrocodylians often have irregular denticles that are not as clearly defined as in *R. sakalavae* (A Martinelli, pers. comm., 2012; Prasad & de Lapparent de Broin, 2002). Baurusuchids and sebecids do have regular denticles, but they are very small in size (Godoy et al., 2014; Marinho et al., 2013; Montefeltro, Larsson & Langer, 2011; Kellner, Pinheiro & Campos, 2013).

The tooth crowns of *Razanandrongobe* bear **transverse undulations** (apicobasal ridges). There are no transverse undulations on the teeth of South American Mesozoic terrestrial crocodylomorphs (A Martinelli, pers. comm., 2012), with the exception of some advanced notosuchia [e.g., *Marialiasuchus*, *Armadillosuchus*, *Caipirasuchus* (Fiorelli et al., 2016)]. Transverse undulations are found in the Jurassic marine crocodylomorph *Dakosaurus* from Patagonia (Gasparini, Pol & Spalletti, 2006), but the premaxilla of this taxon does not match the morphology of that seen in *Razanandrongobe*.

### Phylogenetic affinities

We performed a phylogenetic analysis of *Razanandrongobe sakalavae* among crocodylomorphs, primarily based on the data matrix from Fiorelli et al. (2016) that now includes 113 crocodylomorph taxa and 440 anatomical characters. Following Fiorelli et al. (2016), characters 1, 3, 6, 10, 23, 37, 43, 44, 45,49, 65, 67, 69, 71, 73, 77, 79, 86, 90, 91, 96, 97, 105, 116, 126, 140, 142, 143, 149, 167,182, 187, 193, 197, 226, 228, 279, 339, 356, 357, 364, 368, and 401 were set as additive as they represent nested sets of homologies and/or entail present or absent information.

In order to reduce *a priori* assumptions in the interpretation of some anatomical features of the holotype maxilla, the analysis was performed in subsequent steps. First, *R. sakalavae* was coded according to the unquestionable character states clearly preserved in the known specimens. As the species resulted to be a mesoeucrocodylian nested within Notosuchia, in a second analysis some anatomical features (129, 200, 264, and 396) that were liable to different interpretations were scored following the first phylogenetic result and comparing *R. sakalavae* with the most strictly related taxa. As a consequence, the number of coded characters for *R. sakalavae* increased from 83 to 87.

The character states of *Razanandrongobe sakalavae* in the dataset of Fiorelli et al. (2016) are: 3(1); 4(-); 5(0); 6(0); 7(0); 8(1); 9(0); 13(0); 14(0); 66(1); 77(2); 78(0); 79(1/2); 80(0); 81(0); 103(2); 106(0); 108(0); 119(1); 120(0); 123(0); 124(0); 125(1); 126(0); 127(1); 128(0); 129(0); 135(0); 137(0); 139(1); 140(0); 154(1); 155(1); 158(0); 159(2/3); 161(1); 162(0); 163(0); 164(0); 176(1); 178(0); 183(0); 185(1); 188(0); 189(0); 193(0); 200(0); 213(1); 226(2); 227(1); 231(0); 236(0); 237(0); 239(0); 240(0); 241(0); 242(1); 252(1); 253(0); 262(0); 264(0); 270(0); 283(0); 284(0); 285(0); 286(0); 288(0); 348(0); 363(0); 365(0); 370(0); 381(0); 383(0); 384(0); 385(1); 386(0); 387(1); 388(0); 389(0); 390(0); 392(0); 393(-); 396(1); 400(0); 406(0); 409(0); 410(1).

**Figure 7:**
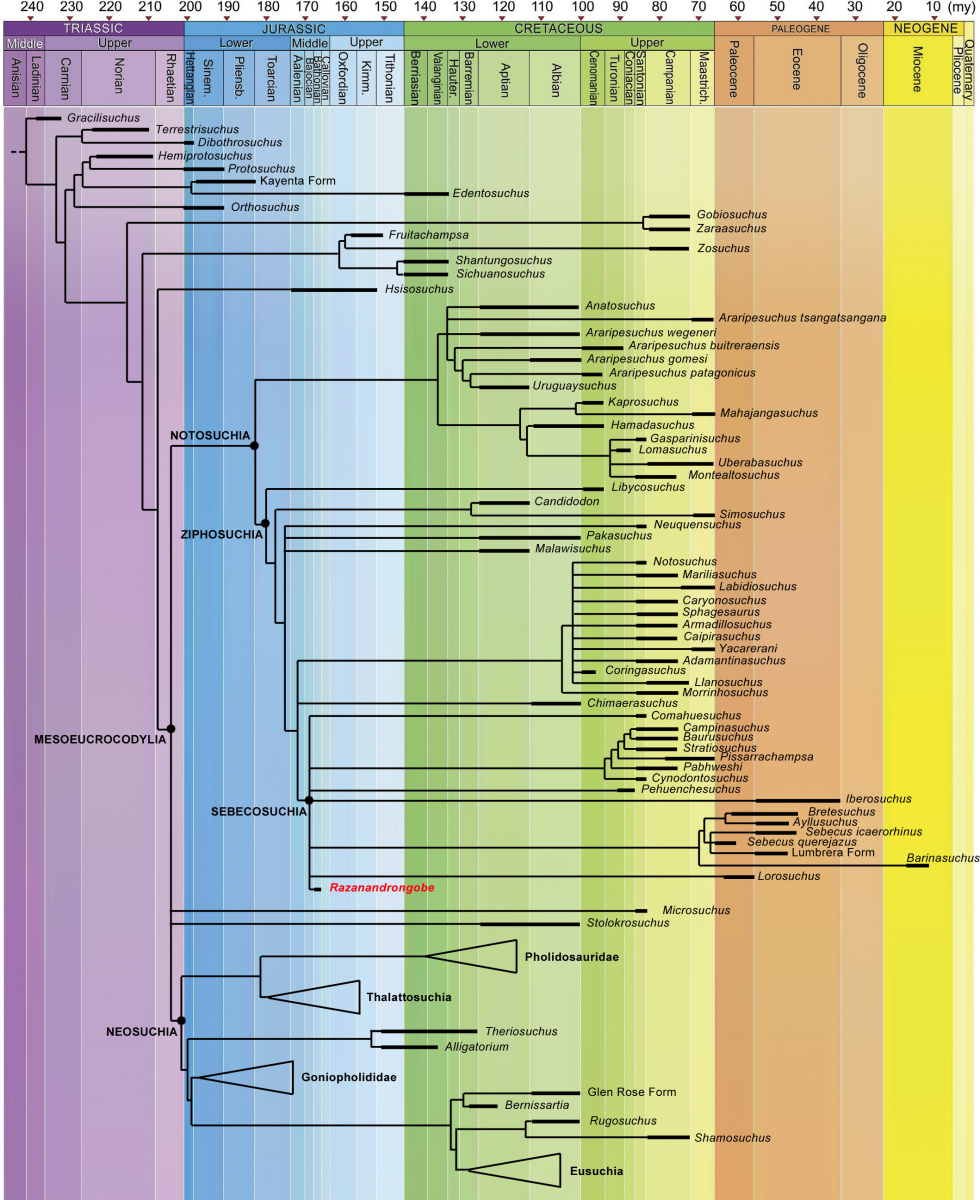
Calibrated phylogenetic relationships of Notosuchia based on the strict consensus topology of 50,000 most parsimonious trees (MPTs). *Razanandrongobe sakalavae* fills a huge gap in the history and diversification of the notosuchian mesoeucrocodylians. Major clades of neosuchians form a polytomy (e.g., Pholidosauridae, Thalattosuchia, Goniopholididae, and Eusuchia) and the age of their oldest member is used to infer the age of these nodes [modified and redrawn from Fiorelli et al. (2016), according to our results]. The complete strict consensus topology is available in Data S3.

**Figure 8:**
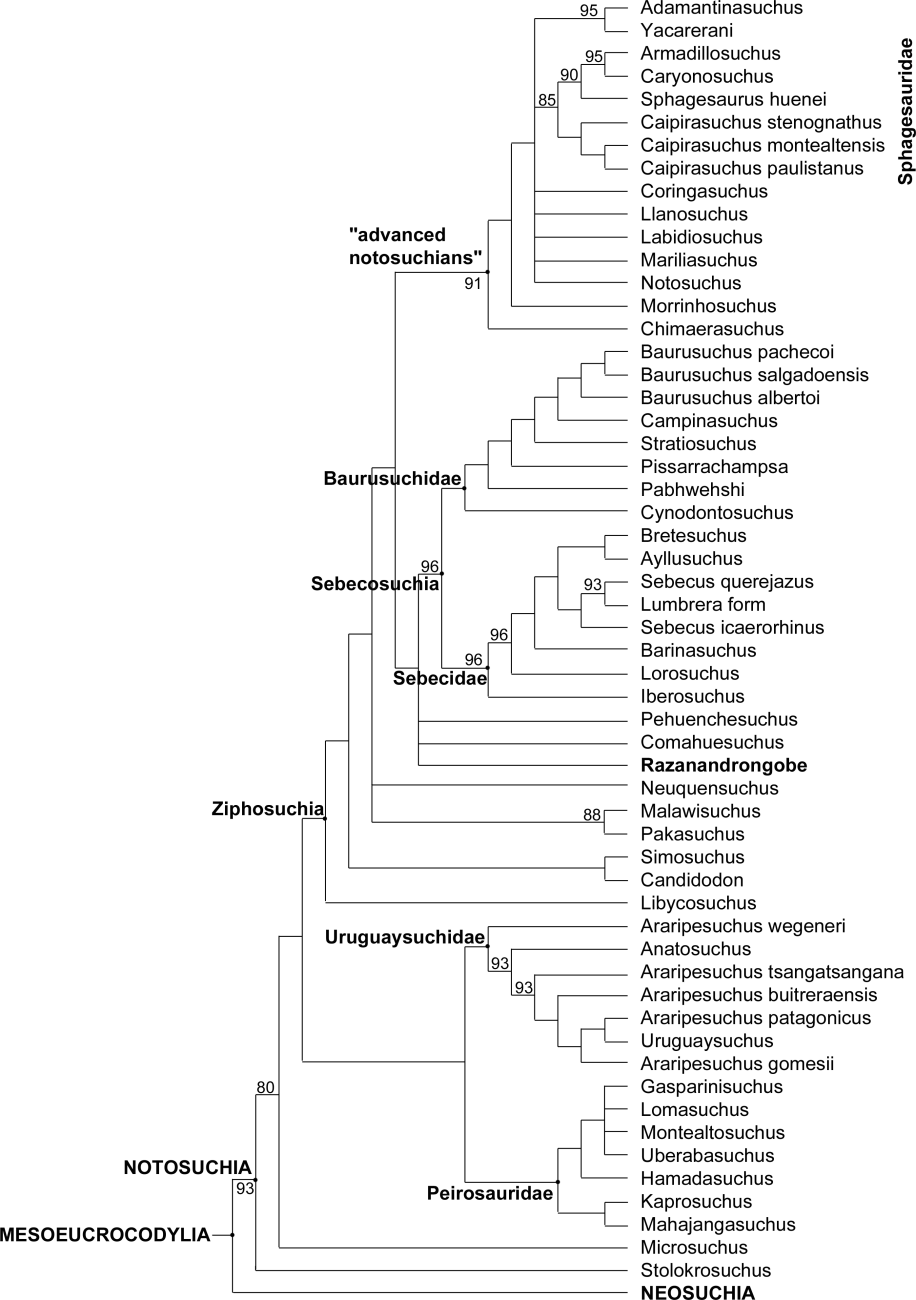
Majority-rule consensus topology of Notosuchia. The 75% majority-rule consensus topology of 50,000 most parsimonious trees (MPTs) generated by PAUP 4.0a150 (Swofford, 2002) on the data matrix in the Supplementary material, showing the phylogenetic relationships of *Razanandrongobe sakalavae* within the Notosuchia. The complete 75% majority-rule consensus topology is available in Data S3.

The complete list of characters from Fiorelli et al. (2016) that can be scored for *R. sakalavae*, along with our comments intended to improve the definition of the character states or to explain the scoring in *R. sakalavae,* is reported in Data S1. The data matrix is available in Data S2. Data S3 includes: settings (1); complete strict consensus of 50,000 trees (2); complete 75% Majority-Rule consensus of 50,000 trees (3); and branch lengths and linkages, apomorphy list, and character change list for Most Parsimonious Tree number 1 under ACCTRAN and DELTRAN transformations (4). The final data matrix was subjected to parsimony analysis with PAUP* 4.0a150. Fifty thousand heuristic replicates (TBR branch swapping on shortest trees, random addition sequence) found 50,000 most parsimonious trees that were 1,738 steps long [consistency index, 0.3038; homoplasy index, 0.6962; retention index, 0.7335; rescaled consistency index, 0.2228—statistics calculated with PAUP (Swofford, 2002)]. The systematic terms are used *sensu* Pol et al. (2014). The obtained topology is similar to that obtained by Pol et al. (2014) and Fiorelli et al. (2016), with all major groups of Notosuchia recovered, i.e., Uruguaysuchidae as the sister group of the Peirosauridae and Mahajangasuchidae; the other notosuchians grouped together in a large and diverse clade (Ziphosuchia) formed by a derived clade of advanced notosuchians (including *Notosuchus* and the Sphagesauridae) and the Sebecosuchia (defined as Baurusuchidae + Sebecidae). *R. sakalavae* resulted in the Mesoeucrocodylia, well-nested within the ziphosuchian notosuchians, and closely related to the Sebecosuchia (Figs. 7 and 8).

The phylogenetic position of *Razanandrongobe* as a ziphosuchian notosuchian more derived than *Malawisuchus* and *Pakasuchus* is supported by two unambiguous synapomorphies: the presence of cheek teeth not constricted at the base of the crown (ch162.0); and a splenial-dentary suture at symphysis that is transversal on ventral surface (ch185.1).

*Razanandrongobe* shares with the Sebecosuchia a mandibular symphysis deep and rostrally convex in lateral view (ch103.2); the presence of denticulate carinae formed by homogeneous and symmetrical denticles with a sharp cutting edge (ch120.0); a dorsal dentary edge with at least a single dorsal expansion and concave posterior to this (ch159.2); and the lateral surface of the dentary below the alveolar margin at mid-region of the toothrow that is vertically oriented, continuous with rest of lateral surface of the dentary (ch193.0). As a terminal taxon, *Razanandrongobe* is characterized by a long list of unambiguous apomorphies: absence of a notch on the premaxilla on the lateral edge of the *aperturae nasi osseae* (ch123.0); nasal lateral edges along the suture with the maxilla nearly parallel (ch128.0); absence of a small neurovascular foramen in the premaxillomaxillary suture on the lateral surface of the rostrum (ch135.0); tooth crowns of the mid-to-posterior regions of the toothrows not compressed laterally, subcircular in cross section (ch140.0); splenial robust dorsally posterior to symphysis, being much broader than the lateral alveolar margin of the dentary at the same region (ch161.1); presence of a wedge-like process of the maxilla on the lateral surface of the premaxillamaxilla suture (ch213.1); circular paramedian depressions present on the premaxillary palate and located rostrally on the premaxilla (ch227.1); ectopterygoid abuts maxillary toothrow (ch264.0); incisive foramen present and large (length equal or more than half the greatest width of premaxillae) (ch285.0); presence of a prominent depression on the palate, near the alveolar margin atthe level of the sixth or seventh alveolus (396.1); and presence of a groove located on the premaxilla lateral surface, running anteroventrally from the dorsoventral midpoint of its posterior margin (ch410.1).

Little is known about the origin and early evolution of the Notosuchia, which are hitherto unknown in the Jurassic. There is a relevant morphological and temporal gap between the basal Crocodyliformes from the Jurassic and the post-Jurassic mesoeucrocodylians, including the notosuchians: as the notosuchians are a sister taxon of the neosuchians, they should have originated at the beginning of the Jurassic, which implies a 74-million-year-long ghost lineage (Fig. 8). According to our phylogenetic results, which is based on robust data and analyses commonly used in studies on fossil crocodylomorphs (Fiorelli et al., 2016; and references therein), *Razanandrongobe* is clearly a Jurassic notosuchian, and by far the oldest representative of the group, predating the other forms by about 42 million years. Thus, it begins to fill the ghost lineage of notosuchians in the Jurassic.

*Razandandrongobe* displays a plesiomorphic morphology that is consistent with its geological age, showing us how limited our knowledge is on the morphology, evolution, and paleobiogeographic history of Notosuchia. Due to the incomplete understanding of our taxon (only 19.8% of the characters scored) and to the scarcity of Middle to Upper Jurassic mesoeucrocodylian taxa recovered to date, the phylogenetic position of *R. sakalavae*, nested deeply inside Ziphosuchia and very close to Sebecosuchia (whose oldest taxon is Santonian), must be regarded as provisional. However, we think that more complete material will only improve the position of *R. sakalavae* within Notosuchia. Likewise, a primarily Gondwanan evolution of the group is supported, but the long (more than 30 million years) ghost lineage undermines any hypothesis on the geographic origin of the group.

### Cranial reconstruction and size

**Figure 9:**
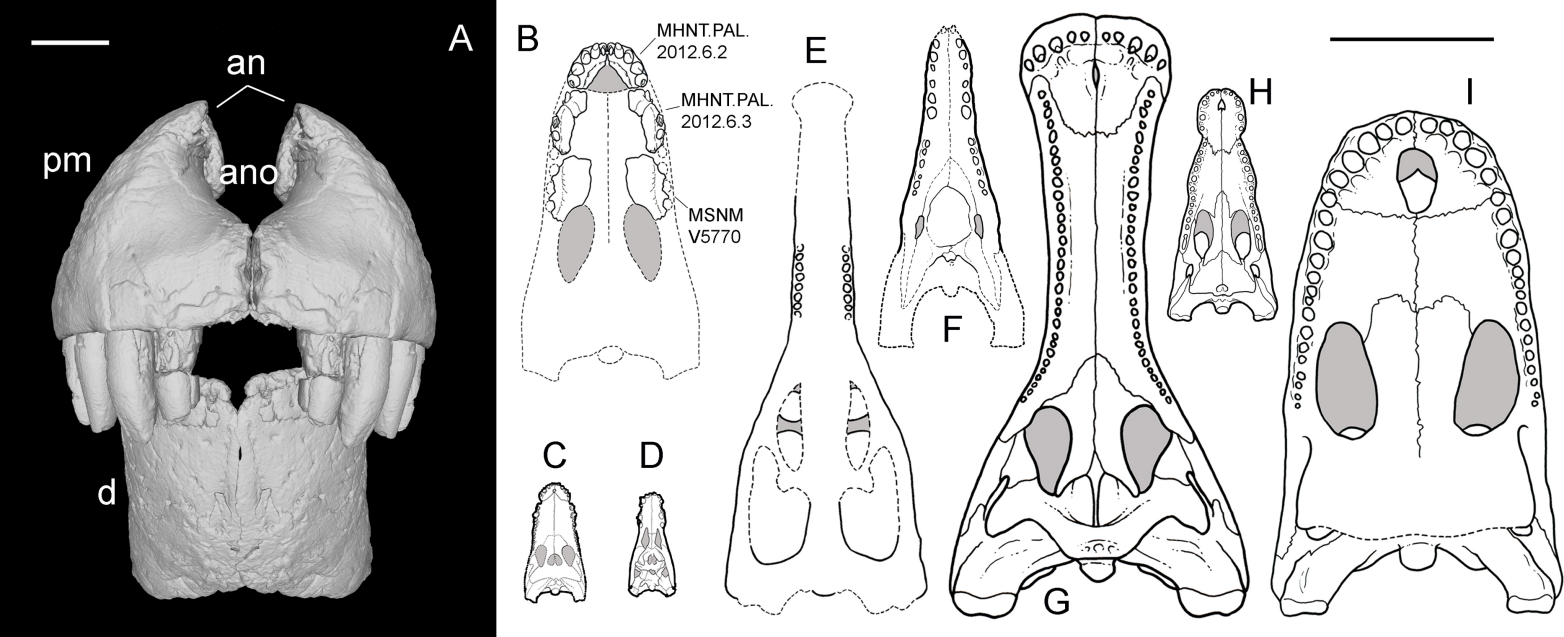
Snout reconstruction and size comparisons. 3-D reconstruction of the snout of *R. sakalavae* in rostral view (A), including the left dentary (MHNT.PAL.2012.0.6.1), the right premaxilla (MHNT.PAL.2012.0.6.2), and their counterlateral copies obtained from CT data; composite reconstruction of the skull of *R. sakalavae* in palatal view ((B) the specimens are indicated in the figure), and size comparisons with the notosuchians *Gasparinisuchus peirosauroides* Martinelli et al., 2012 (C) and* Baurusuchus salgadoensis* Carvalho, De Arruda Campos & Nobre, 2005 (D), the giant thalattosuchian *Machimosaurus rex* Fanti et al., 2016 (E), the giant sebecid *Barinasuchus arveloi* Paolillo & Linares, 2007 (F), the largest extant crocodilian *Crocodylus porosus* Schneider, 1801 (H), and two of the biggest neosuchians, *Sarcosuchus imperator* De Broin & Taquet, 1966 (G), and *Purussaurus brasiliensis* Barbosa-Rodrigues, 1892 (I). Grey areas indicate skull roof areas visible through the palatal fenestrae. Scale bar = 3 cm (A) and 50 cm (B–I). C–I redrawn respectively from: Martinelli et al. (2012), Carvalho, De Arruda Campos & Nobre (2005), Fanti et al. (2016), Paolillo & Linares (2007), Sereno et al. (2001), Grigg & Gans (1993), and Aureliano et al. (2015).

After the phylogenetic analysis, we attempted a cranial reconstruction. It is noteworthy that the size of the new specimens (premaxilla and dentary) well matches that of the holotype maxilla, so no resizing was required to include all specimens in the same drawing. When aligning the premaxillary symphysis to the sagittal plane, the contact surface between premaxilla and maxilla has an incline of 68°. When the rostral right maxillary fragment MHNT.PAL.2012.6.3 is placed in continuity with the premaxilla, it has the suitable curvature (about 21°) to re-align its distal maxillary alveoli to the sagittal plane. In other words, when assembling the premaxilla, the rostral maxillary fragment, and the holotype maxilla (that is likely a mid-caudal portion), we obtain a U-shaped snout when seen in dorsal/ventral views, in which the premaxilla contributes the greatest to the curvature, and the maxilla completes it. Consequently, the snout can be reconstructed as dorsoventrally deep but not mediolaterally narrow, with a rounded tip (Fig. 9). In frontal view, the taller than wide head is somewhat reminiscent of giant aquatic forms such as *Dakosaurus*, other than several notosuchians. The deep jaws, for instance, recall the vertically strong premaxillae, maxillae, and dentaries of baurusuchids and sebecids (see Comparisons within Crocodylomorpha).

*Razanandrongobe sakalavae* might represent the largest non-marine mesoeucrocodylian of the Jurassic, as well as the largest notosuchian, surpassing in size the huge sebecid *Barinasuchus* from the Miocene of Venezuela (Paolillo & Linares, 2007). The alveoli are comparable in size to those of the giant marine thalattosuchian *Machimosaurus rex* (Fanti et al., 2016). The large skull dimensions (and likely body size, too) reached by *R. sakalavae*, together with the peculiar feeding adaptations indicated by the dental and cranial features, suggest that this taxon was a highly specialized predatory crocodyliform of terrestrial habits, that could compete and occupy the ecological niches of theropod dinosaurs. Of note, terrestrial or semi-aquatic species with a skull larger than that of *R. sakalavae* (Fig. 9) and, in all likelihood, with a greater body size, have been found only in more recent strata, e.g., *Sarcosuchus imperator* from the mid-Cretaceous of Niger (Sereno et al., 2001) and the caiman *Purussaurus brasilensis* from the Miocene of Brazil (Aureliano et al., 2015).

## Conclusions

The Middle Jurassic (Bathonian) deposits of the Mahajanga Basin yield a peculiar and poorly known fossil vertebrate fauna. Teeth of thalattosuchian crocodyliforms, plesiosaurs, and possibly ichthyosaurs, as well as dinosaurian remains (mainly sauropods) have been reported over the last century (Thevenin, 1907; Besairie, 1936; Besairie, 1972; Lavocat, 1955; Ogier, 1975; Bonaparte, 1986). Remains of an early tribosphenic mammal (Flynn et al., 1999), pterosaur teeth (Dal Sasso & Pasini, 2003), sauropod teeth and dentary (Buffetaut, 2005), and diverse theropod teeth, vertebrae, phalanges (Flynn et al., 1997; Maganuco, Cau & Pasini, 2005; Maganuco et al., 2007), and track sites (Wagensommer et al., 2011) have also been reported. *Razanandrongobe sakalavae* is the largest terrestrial carnivore from this Middle Jurassic terrestrial ecosystem (Maganuco, Dal Sasso & Pasini, 2006) and was perhaps one of the top predators in Madagascar at the time. Its jaws were extremely robust and high, but possibly short, and bore large teeth with serrated edges resembling those of theropod dinosaurs. Many features of this species strongly suggest that it fed also on hard tissue such as bone and tendon.

The new material described herein has permitted us to score a sufficient number of characters to test the phylogenetic relationships of the species, placing it within the notosuchian mesoeucrocodylians closely related to the sebecosuchians. The phylogenetic analysis also reveals that *R. sakalavae* is a valid species well-distinct from any other currently known member of Notosuchia. In actual fact, it contributes to filling in a gap in the group’s evolution, which contains a long ghost lineage in the Jurassic. It documents a dramatic, somewhat unexpected, size increase in the early history of the group. Moreover, its geographic position during the period when Madagascar was separating from other Gondwana landmasses is strongly suggestive of an endemic lineage. At the same time, it represents a further signal that the Notosuchia originated from southern Gondwana.

##  Supplemental Information

10.7717/peerj.3481/supp-1 Data S1 List of characters coded for *Razanandrongobe sakalavae*

10.7717/peerj.3481/supp-2 Data S2 Data matrix

10.7717/peerj.3481/supp-3 Data S3 Phylogenetic relationships of *Razanandrongobe sakalavae* (MPT1) MPT1 of 50,000 most parsimonious trees (MPTs) generated by PAUP 4.0a150 (Swofford, 2002) on the data matrix in the Supplementary material, showing the phylogenetic relationships of *Razanandrongobe sakalavae* within the Mesoeucrocodylia.

## Institutional abbreviations

MSNM V: Fossil Vertebrate Collection, Museo di Storia Naturale di Milano (Italy)
MHNT. PAL: Paleontological Collection, Muséum d’Histoire Naturelle de Toulouse (France)

## Anatomical abbreviations

a1: first premaxillary/dentary alveolus
a2: second dentary alveolus
am: articular surface for maxilla
an: articular surface for nasal
ano: apertura nasi ossea
apm: articular surface for left premaxilla
asp: articular surface for splenial
d3: third dentary tooth
d7: seventh dentary tooth
df: dental foramina
em: external maxillary wall
etd: edentulous tip of the dentary
g: groove
if: incisive foramen
im: internal maxillary wall
mg: Meckelian groove
mbp: maxillary bony palate
ms: mandibular symphysis
nmp: notch for peg of maxilla
nvf: neurovascular foramina
nvg: neurovascular groove
pade: paramedian depressions for tip of mesialmost dentary teeth
pf: perinarial fossa
pmbp: premaxillary bony palate
pmt: premaxillary tooth
pm5: fifth premaxillary tooth
pspm: paradental shelf of the premaxilla
rt: replacement tooth
vc: vascular canal
ws: wear striations

## Additional Information and Declarations

### Data Availability

The following information was supplied regarding data availability: The raw data has been supplied as a Supplementary File.
