# Supplementary material for: Razanandrongobe sakalavae, a gigantic mesoeucrocodylian from the Middle Jurassic of Madagascar, is the oldest known notosuchian
Source: PeerJ. 2017 Jul 4;5:e3481. doi: 10.7717/peerj.3481 (PMC5499610; doi:10.7717/peerj.3481)
Supplement: Data S1 [file peerj-05-3481-s001.docx]

**Character list -** Here we report the list of characters from Fiorelli et al. (2016) that can be scored for *R. sakalavae* (in **bold**) along with our comments intended to improve the definition of the character states or to explain the coding in *R. sakalavae*. The characters from Turner & Sertich (2010) that, in our opinion, include better definitions and more informative character states with respect to the same characters from Pol et al. (2014) and Fiorelli et al. (2016) are also discussed in the comments below, for future reference. The complete character list is available in Fiorelli et al. (2016); following these Authors, the characters marked with a + were set as additive.

Character 3 (modified from Clark, 1994: char. 3): + Rostrum proportions: narrow oreinirostral (0), broad oreinirostral (**1**), nearly tubular (2), or platyrostral (3).

Comments: the character states (0) and (1) should be quantified; however, according to the condition scored in other taxa we code (1) for *R. sakalavae*.

Character 4 (Clark, 1994: char. 4): Premaxilla participation in internarial bar: forming at least the ventral half (0), or with little participation (1).

Comments: the internarial bar is not present in *Razanandrongobe*, therefore the character is not applicable and must be scored (**-**) as it is the case (for example) in *Sarcosuchus*. This character could be improved adding a character state for the taxa like *R. sakalavae* that lack an internarial bar.

Character 5 (Clark, 1994: char. 5): Premaxilla anterior to nares: narrow (**0**), or broad (1).

Comment: these character states should be quantified.

Character 6 (modified from Clark, 1994: char. 6): + External nares facing anterolaterally or anteriorly (**0**), dorsally not separated by premaxillary bar from anterior edge of rostrum (1), or dorsally separated by premaxillary bar (2).

Character 7 (Clark, 1994: char. 7): Palatal parts of premaxillae: do not meet posterior to incisive foramen (**0**), or meet posteriorly along contact with maxillae (1).

Character 8 (Clark, 1994: char. 8): Premaxilla-maxilla contact: premaxilla loosely overlies maxilla (i.e. posterodorsal process of the premaxilla overlaps anterodorsal surface of the maxilla) (0), or sutured together along a butt joint (**1**).

Character 9 (modified from Clark, 1994: char. 9): Ventrally opened notch on ventral edge of rostrum at premaxilla-maxilla contact: absent (**0**), present as a notch (1), or present as a large notch (2), or present as a notch that is closed ventrally (or largely constrained at its ventral edge) (3).

Character 13 (Clark, 1994: char. 13): Nasal contribution to narial border: yes (**0**), or no (1).

Character 14 (Clark, 1994: char. 14): Nasal-premaxilla contact: present (**0**), or absent (1).

Character 66 (Clark, 1994: char. 66): External nares: divided by a septum (0), or confluent (**1**).

Character 77 (modified from Clark, 1994: char. 77; and Brochu, 1999: char. 43): + Splenial involvement in symphysis in ventral view: not involved (0), involved slightly in symphysis forming up to 20% symphyseal length (1), or forming close to 30% of the symphyseal length (**2**), or extensively involved forming up to 50% of the symphyseal length and occupying more than the length of five alveoli (3).

Character 78 (Clark, 1994: char. 78): Posterior premaxillary teeth: similar in size to anterior teeth (**0**), or hypertrophied (1).

Comments: the modified character 78 of Turner & Sertich (2010) would be more representative of the variability among the taxa. Posterior premaxillary teeth: similar in size to anterior teeth (**0**), or longer but does not form an enlarged caniniform tooth (1), or much longer forming one large premaxillary caniniform tooth (2), or much longer forming two large premaxillary caniniform teeth (3).

Character 79 (modified from Clark, 1994: char. 79): + Enlarged conical maxillary teeth: absent, no tooth size variation (0), one enlarged tooth (or enlarged wave of teeth) (**1**), or enlarged maxillary teeth curved in two waves (festooned) (**2**).

Character 80 (Clark, 1994: char. 80): Anterior dentary teeth opposite premaxilla-maxilla contact: no more than twice the length of other dentary teeth (**0**), or more than twice the length (1).

Character 81 (modified from Clark, 1994: char. 81): Dentary teeth posterior to tooth opposite premaxilla-maxilla contact: equal in size (**0**), or enlarged dentary teeth opposite to smaller teeth in maxillary toothrow (1).

Character 103 (modified from Wu & Sues, 1996: char. 17): Mandibular symphysis in lateral view: shallow and tapering anteriorly (0), deep and tapering anteriorly (1), deep and anteriorly convex (**2**), or shallow and anteriorly convex (3).

Character 106 (modified from Wu & Sues, 1996: char. 27; and Ortega et al., 2000: char.133): Premaxillary teeth: five or more (**0**), four (1), three (2), or two (3).

Comments: the modified character 106 of Turner & Sertich (2010) would be more representative of the variability among the taxa. + Premaxillary teeth: six (0), five (**1**), four (2), three (3), two (4), or one (5).

Character 108 (Wu & Sues, 1996: char. 30): Maxilla: with eight or more teeth (**0**), seven (1), six (2), five (3), or four teeth (4).

Comments: combining information from MSNM V5770 and MHNT.PAL.2012.6.3 we can reconstruct the maxilla as having more than 8 teeth.

Character 119 (modified from Ortega et al., 1996: char. 9): Ventral exposure of splenials along mandibular rami, posterior to the symphysis: absent (0), or present (**1**).

Character 120 (Modified from Ortega et al., 1996: char. 11; Ortega et al., 2000: char. 100; Andrade & Bertini 2008a: char. 132; and Turner & Sertich, 2010: char. 120): Tooth margins in posterior region of the toothrow: with denticulate carinae formed by homogeneous and symmetrical denticles with a sharp cutting edge (**0**), or without carinae or with smooth or crenulated carinae (1), or with tubercular, rounded denticles (anisomorph *sensu* Andrade & Bertini, 2008b) (2).

Character 123 (Pol, 1999a: char. 135): Notch in premaxilla on lateral edge of external nares: absent (**0**), or present on the dorsal half of the external nares lateral margin (1).

Character 124 (Pol, 1999a: char. 136): Dorsal border of external nares: formed mostly by the nasals (**0**), or by both the nasals and premaxilla (1).

Character 125 (Pol, 1999a: char. 138): Posterodorsal process of premaxilla: absent (0), or present extending posteriorly wedging between maxilla and nasals (**1**).

Character 126 (Pol, 1999a: char. 139 and Ortega et al., 2000: char. 9): + premaxilla maxilla suture in palatal view, medial to alveolar region: anteromedially directed (**0**), sinusoidal, posteromedially directed on its lateral half and anteromedially directed along its medial region (1), or posteromedially directed (2).

Character 127 (modified from Pol, 1999a: char. 140): Nasal-premaxilla suture: laterally concave (0), or straight (**1**).

Character 128 (modified from Pol, 1999a: char. 141): Nasal lateral edges along the suture with the maxilla: nearly parallel (**0**), oblique to each other converging anteriorly (1), or oblique to each other diverging anteriorly (2).

Comments: the comparison between the ornamentation and the overall morphology of the specimens MNHT.PAL.2012.6.3 and MNHT.PAL.2012.6.5 indicates that the nasal suture of the latter likely lies on the sagittal plane.

Character 129 (Pol, 1999a: char. 143): Palatine anteromedial margin: exceeding the anterior margin of the palatal fenestrae extending anteriorly between the maxillae (**0**), or not exceeding the anterior margin of palatal fenestrae (1). Comments: Although the palatine is not preserved, this character can be inferred from the maxilla.

Character 135 (Pol, 1999a: char. 149 and Ortega et al., 2000: char. 13): Small neurovascular foramen located in the premaxillo-maxillary suture on the lateral surface of the rostrum (not for large mandibular teeth): absent (**0**), or present (1).

Character 137 (modified from Pol, 1999a: char. 151): Orientation of distal carina on upper posterior teeth and mesial carina on lower posterior teeth: oriented parallel to the longitudinal axis of skull (**0**), or obliquely oriented, at an angle of approximately 45 degrees with the longitudinal axis of the skull (1).

Character 139 (modified from Pol, 1999a: char. 153): External surface of maxilla: with a single plane facing laterally (0), or with ventral region facing laterally and dorsal region facing dorsolaterally (**1**).

Character 140 (Modified from Pol, 1999a: char. 154 and Ortega et al., 2000: char. 104): + Mid to posterior elements of the toothrows: crowns not compressed laterally, subcircular in cross section (**0**), or crowns slightly compressed laterally (1), or roots and crowns highly compressed laterally (2).

Character 154 (modified from Pol, 1999b: char. 212): Shape of dentary symphysis in ventral view: tapering anteriorly forming an angle (0), U-shaped, smoothly curving anteriorly (**1**), or lateral edges longitudinally oriented, convex anterolateral corner, and extensive transversely oriented anterior edge (2).

Character 155 (Pol, 1999b: char. 213): Unsculpted region in the dentary below the tooth row: absent (0), or present (**1**).

Character 158 (modified from Buckley & Brochu, 1999: char. 105): Dentary smooth lateral to seventh alveolus (**0**), or with lateral concavity for the reception of the enlarged maxillary tooth (1).

Character 159 (modified from Ortega et al., 1995: char. 1 and Buckley & Brochu, 1999: char. 107): Dorsal edge of dentary slightly concave or straight and subparallel to the longitudinal axis of skull (0), straight with an abrupt dorsal expansion, being straight posteriorly (1), with a single dorsal expansion and concave posterior to this (**2**), or sinusoidal, with two concave waves (**3**).

Character 161 (modified from Ortega et al., 1995: char. 7 and Buckley & Brochu, 1999: char. 110): Splenial: thin posterior to symphysis (0), or splenial robust dorsally posterior to symphysis, being much broader than the lateral alveolar margin of the dentary at the same region (**1**).

Character 162 (Ortega et al., 1996: char. 13 and Buckley et al., 2000: char. 117): Cheek teeth: not constricted at base of crown (**0**), or constricted (1).

Character 163 (Ortega et al., 2000: char. 10): Ventral edge of premaxilla located: at the same height that ventral edge of maxilla (**0**), or located deeper, with the dorsal contour of anterior part of dentary strongly concave (1).

Character 164 (modified from Ortega et al., 2000: char. 19): Maxillary dental implantation: teeth in isolated alveoli (**0**), or located on a dental groove (1).

Comments: Following Turner & Sertich (2010) we interpret a dental groove to be present when interdental septa are either absent or not visible on the external alveolar margin.

Character 176 (Ortega et al., 2000: char. 101): Width of root of teeth respect to crown: much narrower (0), or subequal or wider (**1**).

Character 178 (Ortega et al., 2000: char. 130): Lateral contour of snout in dorsal view: straight (**0**) or sinusoidal (1).

Character 183 (Ortega et al., 2000: char. 21): Ventral edge of maxilla in lateral view: straight or convex (**0**), or sinusoidal (1).

Character 184 (modified from Ortega et al., 2000: char. 156): Position of first enlarged maxillary teeth: second or third alveoli (0), or fourth or fifth (1).

Comments: In *Razanandrongobe*, all the teeth of the rostral portion of the maxilla are large and subequal in size, as can be seen in the specimen MHNT.PAL.2012.6.3. Therefore, this character cannot be scored in our taxon.

Character 185 (Pol & Apesteguía, 2005: char. 180): Splenial-dentary suture at symphysis on ventral surface: v-shaped (0), or transversal (**1**).

Character 188 (modified from Gomani, 1997: char. 46 and Buckley et al., 2000: char. 113): Cusps of posterior teeth: unique apical cusp (**0**), at least three cusps, a major central cusp with smaller cusps arranged along the mesial and distal margins of the crown (1).

Comments: the modified character 188 of Turner & Sertich (2010) would be more representative of the variability among the taxa. Cusps of teeth: unique cusp (**0**); one main cusp with smaller cusps arranged in one row (1); one main cusp with smaller cusps arranged in more than one row, forming lingual cingulum at base of middle and posterior teeth (2); or multiple small cusps along edges of occlusal surface (3). The morphology previously scored as “several cusps of equal size arranged in more than one row,” which is present only in *Chimaerasuchus paradoxus*, has been removed from the character. We no longer interpret the peculiar morphology of *C. paradoxus* as homologous with the cusps discussed in this character.

Character 189 (Pol & Apesteguía, 2005: char. 184): Dorsal surface of mandibular symphysis: flat or slightly concave (**0**), or strongly concave and narrow, trough shaped (1).

Character 193 (modified from Pol & Apesteguía, 2005: char. 188): + Lateral surface of dentaries below alveolar margin, at mid to posterior region of tooth row: vertically oriented, continuous with rest of lateral surface of the dentaries (**0**), or flat surface facing laterally or laterodorsally but divided by a ridge from rest of the lateral surface of the dentaries (1), or posterior region of alveolar facing dorsally, forming a broad alveolar shelf that is strongly inset medially from the lateral surface of the dentaries (2).

Character 200 (Wu et al., 1997: char. 109): Palatines: form margin of suborbital fenestra (**0**), or excluded from margin of suborbital fenestra (1).

Comments: although the palatines are not directly preserved, their participation to the margin of the suborbital fenestra can be inferred from their contact facets on the maxilla.

Character 213 (Gasparini et al., 1993: char. 3): Wedge-like process of the maxilla in lateral surface of premaxilla-maxilla suture: absent (0), or present (**1**).

Character 226 (Pol & Apesteguía, 2005: char. 221): + Perinarial fossa: restricted extension (0), extensive, with a distinctly concave surface facing anteriorly (1), or large concave surface facing anteriorly, projecting anteroventrally from the external nares and opening toward the alveolar margin (**2**).

Comments: The character from Turner & Sertich (2010) is more adapted to show the variability among the scored taxa, as it includes a fourth state pertaining to the morphology present in peirosaurids. Character 226 (modified from Pol & Apesteguía, 2005: char. 221): + Perinarial fossa: restricted extension (0); extensive, with distinctly concave surface facing anteriorly (1); large concave surface facing anteriorly, projecting anteroventrally from external nares and opening toward alveolar margin (**2**); or extremely large and well-developed, occupying nearly entire surface of premaxilla ventral to external naris (3).

Character 227 (Sereno et al., 2001: char. 67): Premaxillary palate circular paramedian depressions: absent (0), or present and located anteriorly on the premaxilla (**1**). Comments: In *R. sakalavae* these depressions are on the premaxilla close to the pm-m suture. The character 227 from Turner & Sertich (2010) describes this condition better than the character from Pol et al. (2014), thanks to the addition of a character state: Premaxillary palate, circular paramedian depressions: absent (0); present, located anteriorly on premaxilla (1); or present, located at premaxilla-maxilla suture (**2**).

Character 231 (modified from Zaher et al., 2006: char. 195): Procumbent premaxillary alveoli absent (**0**) or present (1).

Character 236 (Pol & Gasparini, 2009: char. 236): Evaginated maxillary alveolar edges: absent (**0**), or present as a continuous sheet (1), or present as discrete evaginations at each alveoli (2).

Character 237 (Pol & Gasparini, 2009: char. 237): Foramen in perinarial depression of premaxilla: absent (**0**), or present (1).

Character 239 (modified from Sereno et al., 2001: char. 68): Premaxillary anterior alveolar margin orientation: vertical (**0**), or inturned (1).

Character 240 (modified from Sereno et al., 2001: char. 69): Premaxillary tooth row orientation: arched posteriorly from midline (**0**); angled posterolaterally, at 120 degree angle (1).

Comments: Turner & Sertich (2010) added a third character state, transverse (2).

Character 241 (Sereno et al., 2001: char. 70): Last premaxillary tooth position relative to maxillary tooth row: anterior (**0**), or anterolateral (1).

Comments: Turner & Sertich (2010) redefined a character state as anterior or anteromedial (0).

Character 242 (Pol & Gasparini, 2009: char. 242): Sutural contact between premaxilla and maxilla on dorsal surface of rostrum posterior to external nares: Premaxillae posterior tip V-shaped, wedging between maxillae (0), or posterior end of premaxillae W-shaped with the anterior tip of maxillae wedging between premaxillae (**1**).

Comments: in Turner & Sertich (2010) the character 242 (Gasparini, Pol & Spalletti, 2006: char. 242) is totally different: Posterior teeth with rings of undulated enamel: absent (**0**), or present (1).

Character 252 (Pol & Gasparini, 2009: char. 252): Sculpture in external surface of rostrum: absent (0), or present (**1**).

Character 253 (Pol & Gasparini, 2009: char. 253): Longitudinal depressions on palatal surface of maxillae: absent (**0**), or present (1).

Character 262 (modified from Brochu, 1997: char. 53): Anterior dentary alveoli project anterodorsally or weakly procumbent (**0**) or strongly procumbent (1).

Comments: Turner & Sertich (2010) separated the two conditions project anterodorsally (0) or weakly procumbent (1), adding the character state (2) for the strongly procumbent condition.

Character 264 (Brochu, 1997: char. 91): Ectopterygoid abuts maxillary toothrow (**0**) or maxilla broadly separates ectopterygoid from maxillary toothrow (1).

Character 270 (Brochu, 1997: char. 153): Incisive foramen completely situated far from premaxillary toothrow, at the level of the second or third alveolus (**0**) or abuts premaxillary toothrow (1).

Comments: Turner & Sertich (2010) modified the character 270 adding a character state: or projects between first premaxillary teeth (or alveolar processes) (2).

Character 283 (Larsson & Sues, 2007: char. 55): Premaxillary teeth 1 and 2, position: separated like adjacent teeth (**0**), or nearly confluent (1).

Character 284 (Larsson & Sues, 2007: char. 60): Large nutrient foramen on palatal surface of premaxillamaxilla contact: small or absent (**0**), or present (1).

Character 285 (Larsson & Sues, 2007: char. 62): Incisive foramen size: present and large (length equal or more than half the greatest width of premaxillae) (**0**), or present or small (1), or absent (2).

Character 286 (Larsson & Sues, 2007: char. 66): Premaxilla-maxilla lateral fossa excavating alveolus of last premaxillary tooth: no (**0**), or yes (1).

Character 288 (Pol & Powell, 2011: char. 288): Nasal exposure on lateral surface of rostrum: deflecting gradually from the dorsal surface (**0**), or deflecting abruptly, forming an almost 90 degree angle between the dorsal and lateral surfaces (1).

Comments: the maxilla fragment MHNT.PAL.2012.6.5 that preserves the articular surface for the nasal indicates that the nasal deflected very little from the dorsal surface.

Character 291 (Pol & Powell, 2011: char. 291): Rugose surface on palatal surface of maxilla posterior to last tooth: absent (0), or present (1).

Comments: in the holotype of *R. sakalavae* the surface posterior to the last preserved tooth of the maxilla is not preserved, therefore the character 291 must be scored (**?**).

Character 348 (modified from Novas et al., 2009: char. 231 by Pol et al., 2014: char. 348): Anterior margin of the suborbital fenestra: maxilla precludes the ectopterygoid-palatine contact at the anterior margin of the suborbital fenestra (**0**), or ectopterygoid projects anteromedially contacting (or almost reaching) the anterolateral end of the palatine, mostly or completely excluding the maxilla from the anterior margin of the suborbital fenestra (1).

Character 363 (Pol et al., 2014: char. 363): Anterior region of dentary symphysis in ventral view: lacking a distinct anterior process, lateral margin of the dentaries diverge gradually (**0**), or having a distinct anterior process with parallel lateral margins (1).

Character 365 (Pol et al., 2014: char. 365): Size of neurovascular foramina on mid to posterior region of alveolar edge of the dentary: small (**0**), or extremely large, being approximately as anteroposteriorly long as an alveolus (1).

Character 370 (Pol et al., 2014: char. 370): Location of the posterior peg in mandibular symphysis: located on the ventral surface of symphysis (**0**), or located above the ventral surface, on the posterior surface of the symphysis (1).

Character 381 (modified from Andrade & Bertini 2008a: char. 128; and Turner & Sertich, 2010: char. 296 by Pol et al., 2014: char. 381): Transitional tooth located at the contact between the premaxilla and maxilla, both of which contribute to the alveolar walls: absent (**0**), or present (1).

Character 383 (Pol et al., 2014: char. 383): Implantation of lower incisiforms: in separate alveoli (**0**), or in a continuous alveolar groove (1).

Character 384 (modified from Andrade et al., 2011: char. 399 by Pol et al., 2014: char. 384): Left and right toothrow along mandibular symphysis: well separated from each other by a broad dorsal surface of the symphysis (**0**), or closely located to each other (forming a symphyseal tooth battery in most taxa) (1).

Character 385 (Pol et al., 2014: char. 385): Apico-basal ridges on the enamel surface of incisiform and caniniform teeth: absent (0), or well-developed (**1**).

Comments: apico-basal ridges on the enamel surface are present at the base of the isolated rostral tooth MSNM V5775 (Maganuco, Dal Sasso & Pasini, 2006) as well as in the large replacement tooth that occupies the first premaxillary alveolus in the specimen MHNT. PAL.2012.6.2.

Character 386 (modified from Andrade and Bertini 2008a: char. 123 by Pol et al., 2014: char. 386): Apicobasal ridges on the enamel surface of posterior teeth: absent (**0**), or present (1).

Character 387 (Pol et al., 2014: char. 387): Separation of apico-basal ridges on the enamel surface of teeth: fine enamel ridges that are closely spaced to each other (flutting) (0), or ridges, usually with a broad base, well-spaced from each other (**1**). Comments: these character states should be quantified.

Character 388 (Pol et al., 2014: char. 388): Size variation of denticles along denticulated carinae: absent or minor variation (**0**), or variable, with denticles at the central region of the carinae being approximately twice the size (height and width) of both apical and basal denticles (1), or decreasing gradually along the carina from the apex to the base of the crown, apical denticles are more than three times the height of the basal denticles (2).

Character 389 (Pol et al., 2014: char. 389): Thin enamel ridge (loph) connecting adjacent denticles instead of presenting distinct interdenticular slits: absent (**0**), or present (1).

Character 390 (modified from Andrade & Bertini, 2008a: char. 149 and O´Connor et al., 2010: char. 233 by Pol et al., 2014: char. 390): Horizontal cingula along the buccal and/or lingual margin of the base of the crown of postincisiform teeth: absent (**0**), or present, with accessory cusps and styli (1).

Character 392 (modified from Turner & Sertich, 2010: char. 294 by Pol et al., 2014: char. 392): Outer enamel surface (between carinae, apicobasal ridges, or flutting, if present): smooth (**0**), rugose (1).

Character 393 (modified from Andrade et al. 2011: char. 374 by Pol et al., 2014: char. 393): Rugose texture on outer enamel surface: formed by anastomizing grooves and ridges (0), formed by small globular protuberances (“pebbled enamel” *sensu* Price, 1950) closely spaced to each other (1). Comments: in *R. sakalavae* most of the crown is smooth, therefore the character must be scored (**-**).

Character 396 (Turner & Buckley, 2008: char. 290): Prominent depression on palate near alveolar margin at level of sixth or seventh alveolus: absent (0), or present (**1**).

Comments: in the holotypic maxilla of *R. sakalavae* a depression on palate near alveolar margin is visible, in a position probably at the level of the alveoli 6-9.

Character 400 (Sereno & Larsson, 2009: char. 83): Single or paired large neurovascular foramina on lateral surface of premaxilla, at its posterolateral corner: absent (**0**), or present (1).

Character 406 (Montefeltro et al., 2011: char. 64): Posteroventral symphyseal depressions: absent (**0**), present (1).

Character 409 (Larsson & Sues, 2007: char. 71): Sagittal torus on maxillary palatal shelves: absent (**0**), or present (1).

Character 410 (Pol et al., 2014: char. 410): Groove located on premaxillary lateral surface, running

anteroventrally from the dorsoventral midpoint of its posterior margin: absent (0), or present (**1**).
